# Cognitive demand as a neurometabolic stress test in diabetes-associated cognitive impairment: from static metabolite pools to dynamic energy responses

**DOI:** 10.3389/fendo.2026.1991245

**Published:** 2026-10-20

**Authors:** Mingyang Li, Min Ye, Hongyu Xie, Ting Gao, Wenjing Kan, Yiming Chen, Yu Xia, Yonghua Zhao, Aihong Yuan

**Affiliations:** 1 The First Affiliated Hospital of Anhui University of Chinese Medicine, Hefei, China; 2 The Second Affiliated Hospital of Anhui University of Chinese Medicine, Hefei, China

**Keywords:** activity-dependent neurometabolic adaptation, brain energy metabolism, cellular substrate allocation, diabetes-associated cognitive impairment, metabolic flexibility, metabolic flux, neurometabolic reserve, neurovascular coupling

## Abstract

**Background:**

Diabetes-associated cognitive impairment (DCI) is heterogeneous and cannot be explained by chronic hyperglycaemia or isolated resting metabolic abnormalities. Measurements of transporter abundance, metabolite pools, perfusion, or functional endpoints alone do not determine whether the brain can dynamically recruit energetic support when cognitive demand increases.

**Objective:**

To conceptualize cognitive demand as a neurometabolic stress test, distinguish static metabolic states from dynamic adaptive responses, and establish a falsifiable framework for activity-dependent neurometabolic failure in DCI.

**Methods:**

This concept-driven critical review used an iterative PubMed/MEDLINE-centred search updated through August 2026, supplemented by backward and forward citation tracking. Evidence was organized along the demand-to-function pathway, encompassing cognitive-task, glucose/insulin, hypoglycaemic or alternative-substrate, and neural/network challenges. Studies were prioritized when they provided information on temporal dynamics, pathway flux, cellular or regional specificity, or causal perturbation.

**Results:**

Normal cognitive function requires coordinated recruitment of vascular delivery, substrate mobilization and allocation, oxidative metabolism, phosphotransfer buffering, and ATP turnover, even when metabolite and high-energy phosphate pools remain relatively stable. Evidence from diabetes and insulin resistance demonstrates heterogeneous, challenge-dependent abnormalities that may involve reduced response amplitude, temporal misalignment, altered substrate distribution, or impaired metabolic utilization rather than uniform cerebral hypometabolism. We propose four non-exclusive within-challenge phenotypes of neurometabolic failure: blunted recruitment, mistimed recruitment, substrate misallocation, and malutilization. Neurometabolic reserve denotes recruitable adaptive capacity; its exhaustion is considered a longitudinal contraction of that capacity, not a fifth within-challenge phenotype or a conclusion warranted by a single attenuated response. However, current evidence has not yet established a complete causal chain linking cognitive demand, substrate fate, energetic support, neural computation, and cognitive performance in clinically characterized DCI.

**Conclusions:**

We propose cognitive demand as an experimental neurometabolic stress test for investigating metabolic vulnerabilities that may remain undetected at rest, rather than as a validated clinical diagnostic test. Activity-dependent neurometabolic failure should therefore be considered a testable contributor rather than an established universal mechanism of DCI. Future studies integrating challenge-locked, temporally aligned, and compartment-resolved metabolic measurements with causal perturbation and longitudinal assessment are required to define when endocrine-metabolic dysfunction translates into cognitive vulnerability.

## Graphical Abstract

**Figure:**
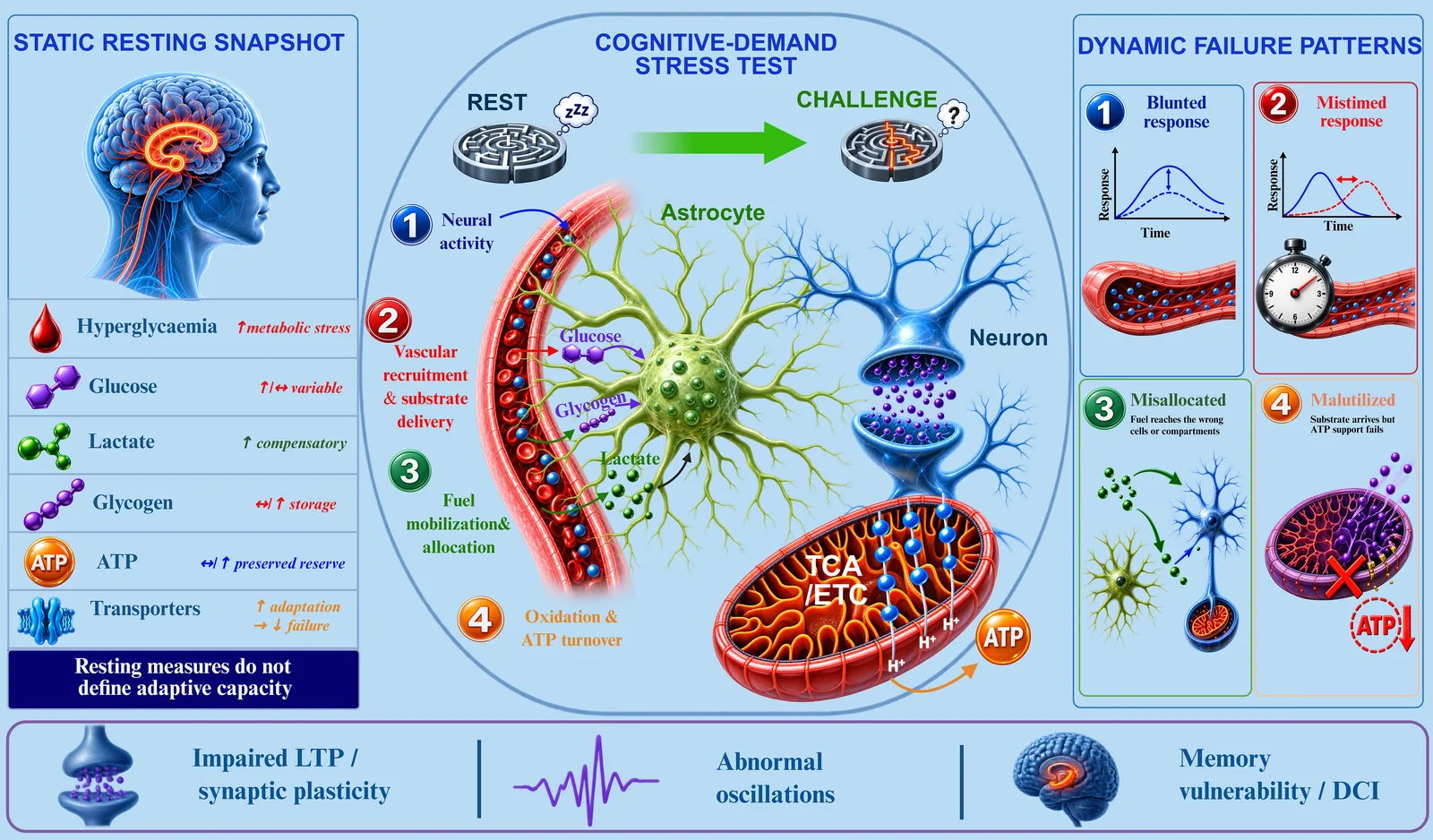

## 1 Introduction

Diabetes is associated with a continuum of cognitive outcomes, ranging from subtle, domain-specific decrements to mild cognitive impairment and dementia. Across prospective studies, diabetes is consistently associated with a higher risk of cognitive impairment and dementia, although the magnitude of that association varies with diabetes type, age, disease duration, comorbidity and outcome definition (1, 2). Longitudinal cohort data further indicate that diabetes in midlife is associated with faster cognitive decline over subsequent decades (3). The phenotype is not confined to memory: executive function, processing speed, attention and psychomotor performance can also be affected, with generally small-to-moderate effects at the group level (4). Accordingly, diabetes-associated cognitive impairment (DCI) is best used as an umbrella term rather than a uniform diagnosis. Its clinical expression probably reflects different combinations of vascular injury, neurodegenerative pathology, metabolic disturbance and reduced cognitive reserve (5, 6). Glycaemic indices are relevant, but they explain only a limited proportion of the observed cognitive variance (7). The central mechanistic problem is therefore not simply whether the diabetic brain has been exposed to abnormal glucose, but why particular circuits become unable to sustain cognition.

Current mechanistic accounts have identified important contributors to DCI, including brain insulin resistance, microvascular dysfunction, blood-brain barrier injury, glial reactivity, neuroinflammation and mitochondrial stress (5, 6, 8). Much of this evidence, however, is derived from measurements obtained at rest or from isolated molecular and biochemical endpoints. Transporter abundance and enzyme expression describe potential capacity; concentrations of glucose, lactate, glycogen, ATP or phosphocreatine describe the size of a pool at a particular time. Neither, by itself, establishes how rapidly substrate is delivered, which cells acquire it, where the carbon is routed, how much is oxidized, or whether ATP production increases when neural work rises. A larger pool may reflect increased production, impaired clearance, reduced uptake or diminished downstream utilization. This ambiguity is not theoretical: during hypoglycaemia, individuals with type 1 diabetes showed markedly increased brain lactate without a corresponding increase in lactate-derived oxidative labelling (9). Static abundance and realized pathway use can therefore diverge. If metabolic vulnerability is expressed mainly when a circuit is challenged, resting snapshots may obscure the biologically relevant phenotype.

Cognitive activity provides a physiologically meaningful perturbation with which to expose that phenotype. In rodents, a hippocampus-dependent spatial task produces a rapid, regionally specific fall in extracellular glucose that scales with task complexity (10). At presynaptic terminals, electrical activity recruits both glycolytic and mitochondrial ATP synthesis, and even brief interruption of this recruitment disrupts vesicle recycling despite a substantial resting ATP pool (11). Learning also mobilizes astrocytic glycogen and lactate transport, while the relative contribution of glucose and lactate varies with the computational and attentional load imposed by the task (12, 13). These cellular responses are embedded within activity-dependent vascular recruitment and rapid neuronal glycolysis (14, 15). Moreover, high-energy phosphate concentrations can remain comparatively stable while ATP-synthesis-related flux changes markedly across levels of brain activity (16). Cognitive demand can therefore be treated as an experimental neurometabolic stress test-not as a validated clinical diagnostic test, but as a controlled means of probing metabolic reserve. In this framework, activity-dependent neurometabolic adaptation denotes the coordinated recruitment of vascular delivery, cellular fuel mobilization and routing, oxidation and phosphotransfer buffering needed to preserve synaptic, network and cognitive function as demand rises.

A challenge-centred view is particularly relevant to diabetes because several studies suggest that basal adequacy can coexist with an abnormal evoked response. In healthy adults, acute lipid infusion used to induce insulin resistance altered the hippocampal phosphocreatine-to-ATP response during sustained cognitive activity (17). This study provides a human proof of principle, but its small sample, acute intervention and ratio-based endpoint do not establish chronic DCI, glucose flux or neuronal ATP deficiency. In rats, high-fat-diet-induced insulin resistance attenuated both the cognitive and local metabolic responses to hippocampal insulin (18). Diet-induced diabetes was also associated with lower hippocampal glycogen and weaker glycogen mobilization after local insulin administration (19). At the vascular level, a mouse model of type 1 diabetes showed delayed stimulus-evoked neurovascular recruitment despite comparatively preserved resting flow and peak responses (20). These observations identify distinct challenge-sensitive abnormalities, but they do not yet form a single causal pathway. They differ in species, diabetes type, disease stage, brain region and experimental challenge; vascular kinetics do not measure substrate flux, and a change in glycogen or high-energy phosphate balance does not by itself establish functional energy failure. No study has yet closed the full chain from cognitive demand to cellular carbon flux, energetic state, neural computation and cognitive performance in clinically characterized DCI.

This Critical Review advances the bounded proposition that impaired activity-dependent neurometabolic adaptation is a testable contributor to DCI. It distinguishes molecular capacity and metabolite pools from task-evoked proxies, pathway flux, energetic state and functional sufficiency. Cognitive, glucose-insulin, hypoglycaemic-substrate and neural-network perturbations are treated as challenge paradigms, not failure phenotypes. The four non-exclusive phenotypes describe how inadequate support may be expressed within a challenge: blunted recruitment, mistimed recruitment, substrate misallocation and malutilization. They are not proposed as new disease mechanisms or discrete DCI subtypes. Their added value is a cross-mechanism, response-based layer of interpretation. Established accounts, including neurovascular dysfunction, brain insulin resistance and mitochondrial impairment, identify candidate lesion classes; the present framework asks how a candidate lesion manifests under demand, at which measured node the abnormality is directly observed, and what additional observation would distinguish competing explanations. It thereby converts broad mechanistic labels into node-specific, falsifiable predictions about response magnitude, timing, carbon allocation and downstream utilisation. Practically, the framework is intended to prevent overinterpretation of a single readout and to specify the next discriminating measurement required to distinguish competing explanations. Reserve exhaustion remains separate and denotes longitudinal contraction of the recruitable response range rather than a fifth acute phenotype. The framework does not assume universal cerebral energy deficiency or one obligatory substrate pathway.

Two examples illustrate how this framework changes interpretation and experimental design. First, a peak-focused analysis can miss temporal dysfunction. In diabetic mice, peak and integrated neurovascular responses can remain comparatively preserved despite delayed recruitment (20), while human studies in type 2 diabetes show altered response rise, peak timing and recovery (21, 22). The framework therefore predicts that a normal or near-normal peak does not exclude a candidate mistimed phenotype and changes the experimental decision from comparing peak amplitude alone to analysing the full response curve in relation to neural timing, using response models flexible enough to capture latency and recovery differences (23). Second, higher insulin-stimulated cerebral FDG uptake reported in insulin-resistant states (24–30) should not be equated with greater oxidative metabolism or successful compensation. FDG primarily reflects delivery, transport and phosphorylation-dependent trapping and does not establish downstream oxidation or ATP support (31–36). Kinetic modelling further suggests that an increased FDG signal can reflect altered inward glucose transfer rather than a proportional increase in downstream metabolism (37). The framework therefore changes the next experimental decision: rather than labelling greater FDG uptake as compensation, the subsequent measurement should target the downstream step required by the specific claim, such as oxidative metabolism or energetic support aligned with neural or cognitive function.

## 2 Literature search and review approach

This concept-driven critical review used iterative PubMed searches, supplemented by backward and forward citation tracking, to identify evidence on activity-dependent neurometabolic adaptation in DCI. Searches combined Medical Subject Headings and title/abstract terms for diabetes or insulin resistance, cognition and hippocampal function, neural activity, neurovascular coupling, substrate metabolism and transport, mitochondrial function, and energetic responses. Complementary searches addressed activity-related diabetes studies, dynamic metabolic measurements, network energetics, human task imaging, substrate transport and reserve capacity, neurovascular and blood-brain barrier function, metabolic interventions, and related reviews. Full search strategies, retrieval counts and evidence-selection criteria are provided in **Supplementary Methods S1**.

Studies were selected for their relevance to the relationships among neural demand, vascular recruitment, substrate delivery and allocation, oxidative metabolism, energetic state, and neural or cognitive function. Priority was given to primary research with clearly described populations or models, brain regions, challenges and measured outcomes, particularly studies providing temporal, pathway-specific, cellular or regional information, or mechanistic perturbation. Countervailing findings and potentially compensatory responses were retained. Physiological and methodological studies established reference mechanisms and measurement limitations; findings outside clinically characterised DCI were interpreted according to their population and experimental context. Static molecular, metabolite-pool or ex vivo capacity measurements informed candidate mechanisms but were not treated as direct evidence of task-evoked metabolic failure.

Evidence was synthesised qualitatively rather than through exhaustive systematic screening. Retrieval limits were applied to three broad searches, which may have restricted literature coverage. Representative primary studies were further evaluated according to their clinical relevance, measurement directness and mechanistic support, with explicit consideration of whether cognitive impairment was characterised in participants with diabetes.

## 3 Cognitive demand as a neurometabolic stress test: the physiological basis

Cognitive demand creates a rapid and spatially localized energetic burden that must be matched within the same challenge window by neurovascular recruitment, fuel mobilization and allocation, oxidative and phosphotransfer responses, and increased ATP turnover (**Figure 1**). Physiological adequacy is defined not by depletion of static metabolite or ATP pools, but by whether energetic support is recruited with sufficient amplitude, timing and spatial precision to preserve synaptic, network and memory function.

**Figure 1:**
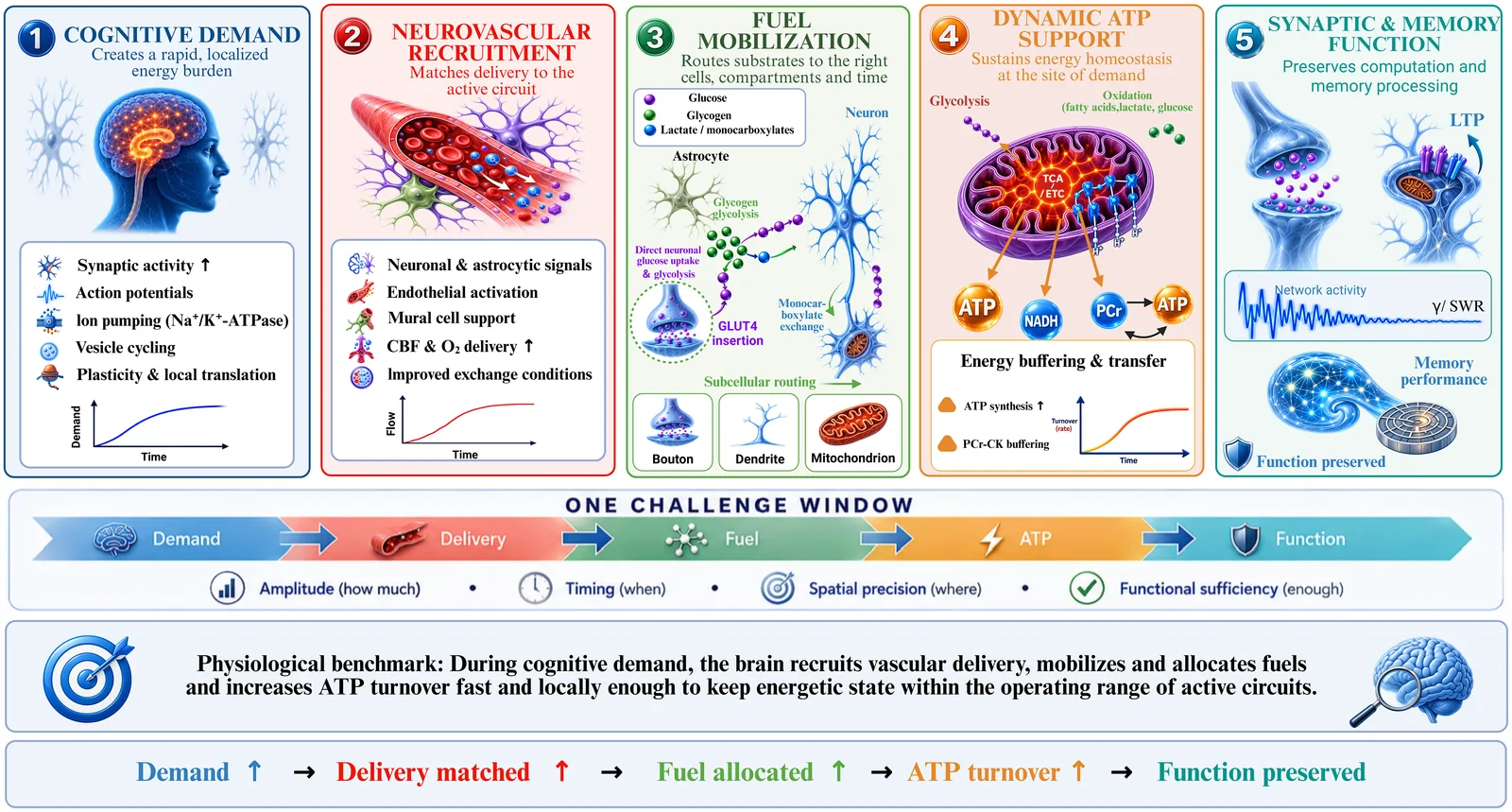
Physiological basis of activity-dependent neurometabolic adaptation during cognitive demand.

Operationally, we use “cognitive neurometabolic stress test” to denote a conceptual and experimental framework, not a validated clinical diagnostic test. Within this framework, a controlled, preferably graded cognitive challenge is paired with measurement of a prespecified vascular or metabolic response from baseline through task performance and recovery. The response should be interpreted alongside achieved performance, systemic physiology and, where feasible, independently quantified neural engagement. The relevant comparison is the response to effective neural demand rather than task exposure alone. The cognitive domain, target circuit, manipulated load and sampling window must therefore be specified. Nominal difficulty does not constitute an equivalent energetic dose across tasks or individuals.

### 3.1 Cognitive demand creates a rapid and spatially localized energy burden

Cognitive demand increases ATP-consuming electrical work within task-engaged circuits. Its magnitude and location depend on which circuit elements carry the computation. Action potentials and postsynaptic currents dissipate Na^+^ and K^+^ gradients that the Na^+^/K^+^-ATPase must restore. Quantitative energy budgets place much of signalling expenditure in postsynaptic conductances and spike propagation, although estimates depend on circuit architecture and modelling assumptions (38, 39). Direct measurements show that action-potential cost varies with neuronal type, membrane state and subcellular location (40, 41). Energetic burden therefore means increased work for local supply and buffering systems, not prior ATP depletion.

Synaptic terminals concentrate this demand. Sustained firing recruits local ATP synthesis, and inhibiting glycolytic or mitochondrial production lowers presynaptic ATP and disrupts vesicle recycling (11). Oxidative ATP is particularly important for endocytosis, although ATP can diffuse along axons to boutons lacking resident mitochondria (42). Vesicular proton leak imposes a substantial resting maintenance cost (43). Mitochondrial pyruvate transport also helps determine presynaptic ATP availability and vesicle-cycle timing (44). The relevant reserve therefore encompasses ongoing maintenance as well as evoked production. These experiments establish a local energetic bottleneck under imposed activity, although they do not directly quantify cognitive-task metabolism *in vivo*.

Excitatory transmission distributes work beyond the firing neuron. Na^+^-coupled glutamate uptake increases ionic pumping and glycolytic demand in astrocytes (45). Neuronal stimulation can simultaneously accelerate neuronal glucose consumption and glycolysis (15). This distributed workload supports transmitter clearance and ionic homeostasis around active synapses. Active circuits therefore recruit parallel cellular responses whose relative contributions vary with preparation, stimulus and time scale. Opposing local changes may be obscured in bulk tissue averages.

Memory formation adds biosynthetic demand. Protein synthesis and structural remodelling extend that demand beyond the initial electrical event. Stable dendritic mitochondrial compartments support local translation and spine growth, and their disruption impairs plasticity within nearby dendritic segments (46). Learning also depends on hippocampal monocarboxylate metabolism, protein synthesis and persistent plasticity in intervention-rescue models (12, 47). These findings locate the relevant metabolic burden near modified synapses without establishing a single obligatory route of substrate transfer.

Cognitive tasks show that this burden is graded and regionally restricted. Task difficulty can therefore operate as a graded metabolic challenge. During maze learning, extracellular hippocampal glucose declined more during the complex spatial condition and remained unchanged at a non-hippocampal control site (10). The concentration change reflects delivery and removal jointly, so it cannot identify uptake or oxidation. Chronic neuronal loss of glucose uptake or glycolysis also compromises presynaptic ATP and age-dependent learning and memory (48). Developmental compensation, however, limits task-locked causal interpretation.

At the systems level, isotope tracing links graded glutamatergic activity to oxidative glucose metabolism (49). Magnetization-transfer studies likewise show activity-dependent changes in cerebral ATP-synthesis-related flux despite comparatively stable high-energy-phosphate concentrations (16). Although neither experiment used a cognitive task and both require model-based interpretation, they define the physiological problem. Demand must recruit delivery, fuel handling and ATP turnover at the relevant time and place without necessarily depleting ATP pools. The defining feature is dynamic recruitment, rather than a large change in stored energy.

### 3.2 Neurovascular recruitment matches substrate and oxygen delivery to neural activity

Neurovascular coupling (NVC) converts neural activity into regional vascular responses, principally functional hyperaemia (14, 50). By increasing local perfusion, it improves access to oxygen and blood-borne substrates within task-engaged tissue. This distributed, time-varying response changes exchange conditions at the neurovascular interface. It thereby establishes supply capacity before cells select and metabolise fuels. It does not imply fixed proportionality among cerebral blood flow (CBF), substrate transport, oxygen consumption and ATP production.

Functional hyperaemia arises from convergent cellular signals rather than a single neuron-astrocyte-vessel relay. Excitatory, inhibitory and nitric oxide synthase-expressing neurons produce distinct vascular effects (51, 52). Sensory dilation can occur without canonical IP3R2-dependent astrocytic calcium signalling (53). Fine-process astrocytic calcium can nevertheless precede olfactory hyperaemia (54). Calcium clamping preferentially attenuates the late response to prolonged stimulation (55). Astrocytic effects may also differ between capillaries and arterioles (56). Their contribution is therefore vessel-, region- and duration-dependent. This heterogeneity becomes especially important as the duration of cognitive demand changes.

The vascular tree further distributes recruitment across endothelial and mural-cell mechanisms. Capillary endothelial K+ sensing can propagate hyperpolarisation towards upstream arterioles (57). Endothelial disruption interrupts stimulus-evoked dilation (58). Arteriolar endothelial caveolae support dilation and capillary red-cell acceleration in awake mice (59). Reports differ on whether early capillary dilation reflects pericyte relaxation (60). Vessel-zone mapping instead assigns the fastest diameter changes to arterioles and ensheathing mural cells (61). Thin-strand pericytes can alter local resistance more slowly (62). Whole-arbor imaging confirms segment-specific diameter and velocity responses (63). Fast upstream control can therefore coexist with slower capillary resistance adjustments. Functional hyperaemia is a coordinated vascular-tree response rather than a unitary event.

Flow recruitment improves delivery but does not specify metabolic use. Somatosensory activation can increase CBF much more than cerebral oxygen consumption (64). Visual stimulation similarly produces larger flow and glucose-utilisation responses than oxygen-metabolic responses (65). Across graded visual stimuli, CBF and CMRO_2_ may covary linearly, although fractional flow changes remain larger and paradigm-specific (66). CBF is accordingly a supply-side readout rather than a direct measure of use. Delivery, extraction, transport, uptake and oxidation are consecutive but non-equivalent processes.

Timing is equally important. Haemodynamic responses begin within the parenchyma and propagate along selected arterioles with compartment-specific onset and recovery (67). Much vessel-resolved evidence comes from sensory preparations, leaving hippocampal cognitive recruitment less defined. BOLD responses track local field potentials more closely than spiking in visual cortex, with site-specific transfer functions (68). BOLD therefore reports vascular and oxygenation consequences of local processing rather than neural activity or metabolic flux directly (69). Temporal alignment must be evaluated against the neural event that generated the response.

Neurovascular recruitment is thus necessary but insufficient for matching demand. Preserved CBF or BOLD may coexist with impaired barrier transport, cellular allocation, oxidation or ATP support. Modest haemodynamic changes may instead be offset by extraction or local buffering. A normal peak alone does not establish timely supply. Adequacy depends on amplitude, latency, duration and spatial reach within the relevant activity window. The next question is how delivered substrates are allocated to active cells and compartments.

### 3.3 Cellular and subcellular fuel mobilization supports memory processing

Once substrates reach the neurovascular interface, allocation becomes decisive. Delivery alone does not determine cellular uptake, glycogen mobilisation or carbon transfer to active boutons, dendrites and mitochondria. Current evidence supports concurrent neuronal glucose use, astrocytic carbohydrate metabolism and monocarboxylate exchange, with contributions varying by region, workload and memory phase (70). Fuel mobilisation is therefore a kinetic and compartmental response rather than a change in bulk metabolite abundance.

Neurons can rapidly recruit glucose. Stimulation increases neuronal uptake of a 2-deoxyglucose analogue in awake mice (71). Genetically encoded sensors identify an early neuronal glycolytic response that does not require lactate uptake within the sampled interval (15). Conditional loss of neuronal GLUT3 or PKM1 impairs hippocampal energetic state and learning despite metabolic remodelling (48). At boutons, AMPK-dependent GLUT4 insertion sustains vesicle recycling during prolonged firing (72). These models establish direct neuronal recruitment without excluding parallel astrocytic or later monocarboxylate support.

Astrocytes provide complementary support while meeting their own activity-dependent workload. Glutamate uptake stimulates astrocytic glycolysis and lactate production (45). Hippocampal activation recruits glycolytic and oxidative responses in both astrocytes and neurons (73). Astroglial networks can redistribute glucose-related metabolites when extracellular glucose is limited (74). Glycogen supplies a rapid reserve. Blocking glycogenolysis during memory tasks impairs performance that an oxidisable substrate can rescue (75). Disrupting glycogen breakdown or monocarboxylate transport also impairs long-term memory and persistent potentiation (12). Deleting brain glycogen synthase weakens learning-associated CA3-CA1 plasticity (76). These interventions establish functional importance without identifying the final recipient or oxidative fate of glycogen-derived carbon.

Lactate is therefore a conditional fuel and signalling route, not an obligatory endpoint. Cell-resolved sensing reveals an *in vivo* gradient compatible with astrocyte-to-neuron transport (77). Dynamic ^13^C magnetic resonance spectroscopy shows that circulating lactate can contribute carbon to human brain oxidation when plasma availability rises (78). Lactate also modifies neuronal redox state and NMDA receptor-dependent plasticity-gene expression (79). Lactate, pyruvate and beta-hydroxybutyrate can each rescue selected translation or memory deficits (47). Such flexibility, together with bidirectional monocarboxylate transport, prevents transporter abundance from specifying carbon-flow direction.

Allocation extends below the cellular level. Activity-dependent GLUT4 trafficking positions glucose entry at presynaptic boutons (72). Stable dendritic mitochondrial domains support translation and spine enlargement within restricted segments (46). Their local disruption can impair nearby plasticity despite apparently preserved cell-wide metabolism. Transporter localisation and mitochondrial proximity therefore define potential access to fuel and ATP support, but neither alone identifies the oxidised substrate or the energy supplied to an engaged synapse.

Activity-dependent fuel mobilisation is organised by substrate source, recipient, timing and metabolic fate. Neuronal glucose use and astrocytic glycogen-monocarboxylate support may contribute concurrently and shift across acquisition, consolidation and sustained network activity. Assigning carbon flow to specific cells and subcellular domains in the behaving brain remains difficult (80). Mobilisation is physiologically adequate only when routed carbon supports the ATP response required by the relevant computation.

### 3.4 Dynamic ATP production preserves energetic and functional homeostasis

During neural activity, energetic homeostasis can be preserved without increasing the ATP pool. *In vivo* magnetization transfer shows that ATP-synthesis-related and creatine-kinase exchange fluxes change while high-energy-phosphate concentrations remain comparatively stable (16). At boutons, stimulation recruits glycolytic and mitochondrial ATP production that limits presynaptic ATP decline (11). Cytosolic ATP and ADP may remain stable across physiological workloads (81). Larger sodium loads can instead produce delayed somatodendritic ATP falls (82). Physiological-like firing may also cause small decreases at specialised terminals (83). These observations define a load-, time- and compartment-dependent energetic margin.

ATP support draws on coordinated pathways rather than a fixed substrate hierarchy. White-matter ATP dynamics track electrical conduction and local metabolic support (84). Oxidative phosphorylation can rapidly power hippocampal presynaptic and postsynaptic processes (85). Glycolysis or respiration may temporarily sustain basal transmission, whereas recovery from intense vesicle depletion depends more strongly on oxidative metabolism (86). Neurons also accelerate glycolysis during stimulation (15). Mitochondrial calcium uptake promotes NADH generation and tricarboxylic-acid-cycle recruitment (87). MICU3 increases axonal mitochondrial calcium sensitivity, enabling activity-dependent ATP synthesis from oxidative fuels (88). Pathway contributions consequently vary by workload, substrate environment and compartment.

Buffering and spatial organisation extend this margin. Axonal ATP diffusion can support boutons without resident mitochondria, although endocytosis becomes vulnerable when oxidative supply is constrained (42). Brain-type creatine-kinase deletion preserves ATP and phosphocreatine concentrations but reduces phosphocreatine-ATP exchange and slows spatial learning (89). Syntaphilin-dependent mitochondrial docking regulates presynaptic calcium dynamics and short-term facilitation (90). Stable dendritic mitochondrial domains support local translation and spine plasticity (46). These effects cannot be assigned solely to ATP because mitochondria also regulate calcium and redox signalling.

Functional impairment may emerge before global ATP collapse. Local interruption of ATP production rapidly disrupts vesicle endocytosis, ion-gradient restoration and sustained transmitter cycling (11, 42). Long-term potentiation recruits dendritic mitochondrial fission and matrix calcium, and blocking this response impairs structural and electrophysiological plasticity (91). Hippocampal gamma oscillations require strong complex-I performance (92). Oxygen use differs among gamma activity, sharp-wave ripples and lower-demand states (93). Reducing the mitochondrial calcium uniporter disrupts gamma synchrony, sharp-wave ripples and activity-dependent pyruvate-dehydrogenase regulation (94). Together, these studies link mitochondrial recruitment to demanding network states without attributing every functional change solely to ATP loss.

At larger scales, increased ATP-synthesis-related flux can coexist with buffered ATP and phosphocreatine pools (16). A local ATP decline instead indicates that consumption has temporarily exceeded synthesis, redistribution or buffering (82, 83). Neither pattern is inherently pathological. Interpretation depends on challenge intensity, duration, compartment and sampling time. Redox, oxygen-consumption, phosphocreatine-exchange and ATP signals describe different response components, while preserved output may reflect compensation or a lower effective workload.

Normal neurometabolic adaptation therefore requires glycolytic, mitochondrial and phosphotransfer processes to increase turnover with sufficient amplitude, timing and spatial precision to keep the active circuit within its energetic operating range. Adequacy is established by preservation of the relevant synaptic or network function, not by a single ATP concentration or substrate measurement. This demand-matched response provides the physiological benchmark against which diabetes-related abnormalities should be judged.

## 4 From static pools to dynamic responses: a measurement-to-inference framework

Neurometabolic evidence spans molecular capacity, metabolite pools, task-evoked proxies, tracer- or model-derived rates, energetic state and functional outcomes (**Figure 2**). These domains are complementary but not interchangeable. **Table 1** summarises what each major readout measures or estimates, what it cannot establish alone and its principal interpretive limitations. Cross-level claims require measurements aligned in time, compartment and challenge context.

**Figure 2:**
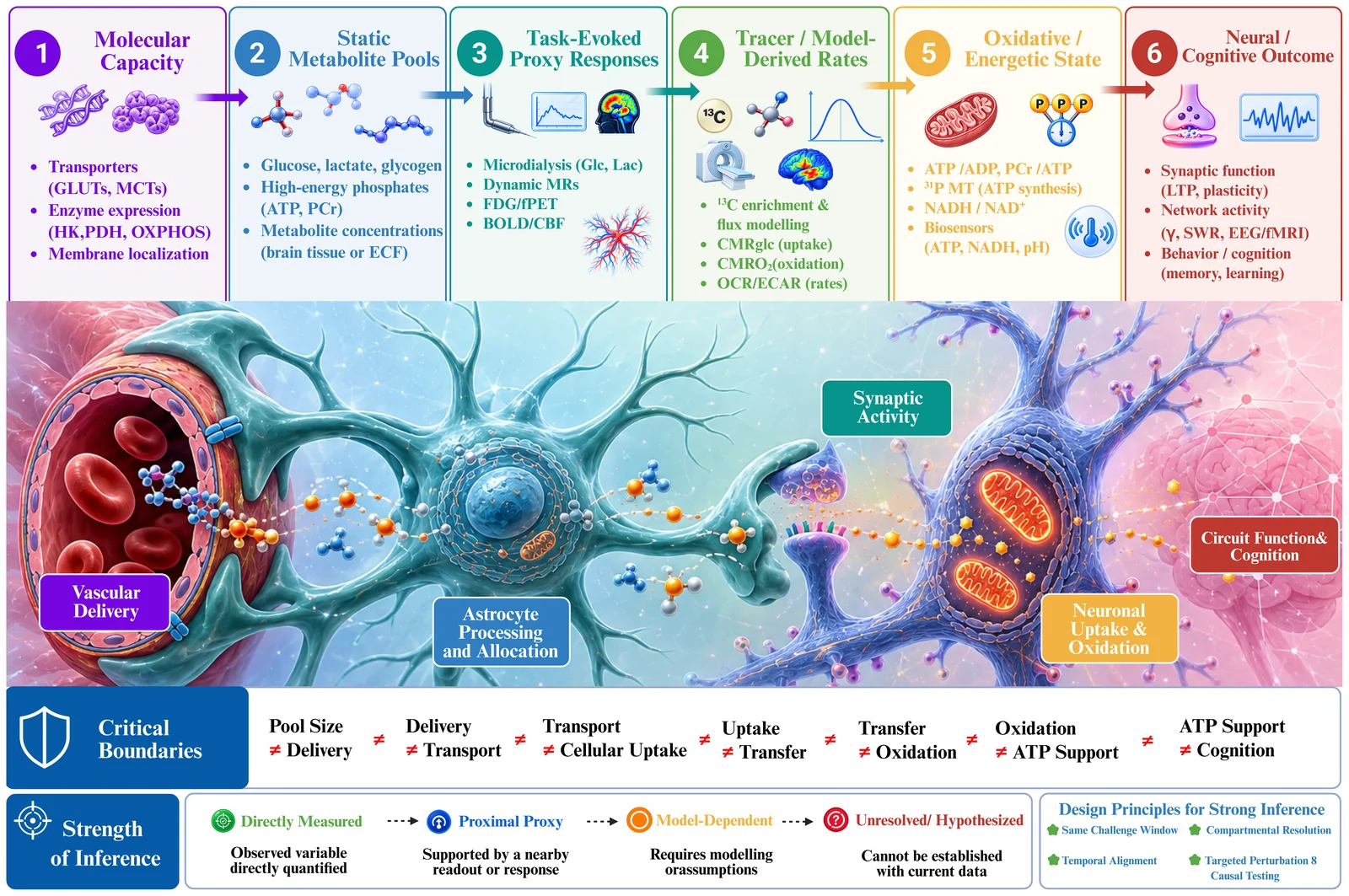
A measurement-to-inference framework for evaluating neurometabolic evidence in diabetes-associated cognitive impairment.

**Table 1:** What common neurometabolic measurements establish and their principal interpretive limitations.

Measurement/readout	What it measures or estimates	What it does not establish on its own	Principal interpretive limitations
Molecular abundance and ex vivo enzyme capacity	Transporter, enzyme, transcript or protein abundance indicates potential molecular capacity, while ex vivo activity assays define capacity under the imposed assay conditions (80).	These measurements do not establish realised *in vivo* transport, pathway throughput, task-evoked flux or adequate ATP support (80, 95, 96).	Interpretation depends on cellular and membrane localisation, substrate and cofactor supply, saturation, regulation, compartmentation and assay conditions (80, 95, 96).
Metabolite concentrations and pool dynamics (tissue assays, microdialysis, ¹H MRS)	They quantify metabolite abundance or concentration in a sampled tissue, voxel or extracellular compartment; serial measurements describe changes in pool size over time (10, 80, 97, 98).	Concentration alone does not separate delivery, production, consumption or export, and does not establish absolute uptake, oxidation or ATP production (80, 95, 96).	The same pool size can arise from different turnover rates; spatial averaging and sampling interval can obscure compartment-specific or rapid changes (80, 95, 96).
FDG-PET/FDG-fPET	FDG signals integrate tracer delivery, transport and hexokinase-dependent phosphorylation/trapping; with appropriate kinetic modelling, net influx or glucose utilisation can be estimated (31–33).	FDG does not directly establish downstream glycolytic flux, TCA-cycle oxidation, ATP synthesis or neuron-specific glucose use (31, 34).	Interpretation depends on the reported metric, input function, plasma glucose, lumped constant, acquisition window and temporal filtering; astroglial processes can contribute to the signal (32–36).
CBF/perfusion measurements (ASL, H_2_¹^5^O PET; TCD for arterial velocity)	ASL and H_2_¹^5^O PET estimate regional perfusion, whereas transcranial Doppler measures arterial blood-flow velocity rather than tissue perfusion (64–66, 99, 100).	Perfusion alone does not establish substrate extraction, transendothelial transport, cellular uptake, oxidation or adequate ATP support (64–66, 99).	Flow can dissociate from glucose and oxygen metabolism, and interpretation is affected by vascular tone, systemic physiology and the measurement modality (64–66, 99, 100).
Conventional BOLD-fMRI	BOLD reflects deoxyhaemoglobin-sensitive haemodynamic changes associated with local neural processing (68, 69).	BOLD is not an independent quantitative measure of neural firing, CBF, CMRO_2_, glucose metabolism or ATP production (68, 69).	Flow, blood volume, oxygen extraction, baseline vascular physiology and neurovascular coupling jointly shape BOLD amplitude and kinetics (68, 69, 101).
Stable-isotope tracing and dynamic ¹³C MRS	These approaches track label incorporation and substrate contribution; with precursor input and kinetic modelling, specified pathway or compartmental rates can be estimated (95, 96, 102–106).	Isotopic enrichment alone does not establish absolute net flux, direct cell-to-cell transfer or ATP output (95, 96, 102–107).	Precursor enrichment, pool size, unlabelled dilution, reversible exchange, competing pathways, acquisition duration and model structure determine the inference (95, 96, 102–106).
Hyperpolarised ¹³C MRI/MRS	Hyperpolarised tracers provide rapid regional substrate-conversion or label-exchange signals and can yield kinetic or redox-related information when the tracer and model permit (108).	A conversion signal alone does not establish net glycolytic or oxidative flux (108).	Delivery, transport, reversible exchange, redox state, polarisation decay and model assumptions contribute to the observed signal (108).
CMRO_2_ (¹^5^O-gas PET or calibrated MR estimates)	CMRO_2_ quantifies or estimates cerebral oxygen consumption within an explicit physiological model (109–112).	CMRO_2_ does not identify the oxidised substrate or consuming cell type and does not directly measure total ATP production (110–112).	Flow/oxygenation calibration and modelling remain part of the estimate; conversion to ATP output assumes coupling, an effective P/O ratio and limited non-mitochondrial oxygen use (110–112).
Respirometry and extracellular-flux assays	These assays quantify oxygen-consumption and acidification responses and can derive ATP-linked respiration, proton leak, maximal respiration, spare capacity and corrected ATP-production estimates (113–115).	They do not by themselves establish intact-brain ATP support during cognition or longitudinal neurometabolic reserve (113–115).	Uncoupling, non-mitochondrial oxygen use, respiratory CO_2_, substrate conditions and stoichiometric corrections influence derived quantities; ex vivo capacity is context dependent (113–115).
High-energy-phosphate pools and ratios (ATP, PCr, Pi; PCr/ATP by ³¹P MRS)	Conventional ³¹P MRS measures high-energy-phosphate abundance, intracellular pH and relative pool balance; PCr/ATP is a pool ratio (16, 17).	Static pools or ratios do not establish ATP synthesis or turnover rate, preserved neurometabolic reserve, or adequate ATP support of active synapses (11, 16, 17).	Buffering and spatial averaging can maintain bulk ATP or PCr despite altered local synthesis, consumption, redistribution or turnover (11, 16).
³¹P magnetisation transfer	Magnetisation-transfer approaches provide model-dependent estimates of unidirectional phosphate-exchange fluxes within the ATP-PCr-Pi network (16, 116).	The measured exchange does not necessarily equal net oxidative ATP synthesis unless the relevant biochemical and kinetic assumptions are validated (116, 117).	Reversible near-equilibrium exchange and model assumptions can separate measured exchange from net ATP production; interpretation is tissue- and protocol-specific (116, 117).
ATP/ADP and redox biosensors	Genetically encoded sensors can resolve local or subcellular ATP-to-ADP or NADH-to-NAD^+^ dynamics with high temporal resolution (118–120).	These signals do not by themselves quantify absolute pathway flux or identify the provenance of metabolised carbon (118–120).	Affinity, saturation, pH, expression, calibration and targeting affect signals that integrate production, consumption, transport and buffering (118–120).

ASL, arterial spin labelling; ATP, adenosine triphosphate; BOLD, blood oxygen level-dependent; CBF, cerebral blood flow; CMRO_2_, cerebral metabolic rate of oxygen; FDG, fluorodeoxyglucose; fPET, functional PET; MRS, magnetic resonance spectroscopy; PCr, phosphocreatine; PET, positron emission tomography; Pi, inorganic phosphate; TCA, tricarboxylic acid cycle; TCD, transcranial Doppler.

### 4.1 Molecular capacity and static metabolite pools

Transporter or enzyme abundance and metabolite concentrations define molecular capacity and pool state rather than realised metabolic throughput (**Table 1**). *In vivo* pathway activity additionally depends on cellular localisation, substrate and cofactor supply, compartmentation and downstream use (80, 95, 96). Diabetes illustrates this pool-flux dissociation: GLUT3 abundance can increase despite lower glucose utilisation (121), brain glycogen can remain relatively preserved despite slower ^13^C incorporation (122), and a larger lactate pool during hypoglycaemia need not be accompanied by greater downstream oxidative labelling (9). High-energy-phosphate pools are similarly buffered, because ATP and phosphocreatine may remain comparatively stable while activity-related exchange or synthesis-related flux changes (11, 16). Static abundance should therefore be treated as a constraint or hypothesis-generating signal rather than direct evidence of impaired delivery, oxidation or ATP support.

### 4.2 Task-evoked concentration, uptake, and haemodynamic responses

Task-evoked concentration, uptake and haemodynamic responses can reveal abnormalities hidden at rest, but each remains pathway-specific (**Table 1**). Microdialysis and ^1^H MRS report concentration balance rather than uptake or oxidation, FDG integrates delivery, transport and phosphorylation-dependent trapping, and CBF or BOLD characterises vascular or haemodynamic responses rather than downstream ATP production (10, 31–36, 64, 65, 68, 69, 97–101). Multimodal dissociation is therefore informative rather than contradictory: FDG and BOLD responses can diverge within the same task (35, 36).

In diabetes, memory-task FDG responses may be spatially diffuse (123), cognitive-task cerebrovascular recruitment can be reduced despite similar resting flow velocity (100), hypoglycaemia can redistribute perfusion (124), and adjustment for cerebrovascular reactivity can alter apparent BOLD associations (101). Preclinical models likewise show smaller or delayed evoked vascular responses (20, 125). These observations identify uptake or vascular-response abnormalities but do not establish the complete pathway from cognitive demand to oxidative ATP support.

### 4.3 Tracer-derived flux, oxidative metabolism, and cellular energetic state

Tracer and energetic methods resolve deeper steps of the pathway but remain model-dependent (**Table 1**). Isotopic enrichment is not equivalent to flux unless precursor input, pool size, sampling kinetics and an explicit model are incorporated. Hyperpolarised ^13^C improves temporal sensitivity but remains influenced by delivery, exchange, redox state and polarisation decay (108) (97). CMRO_2_ more directly addresses oxidative demand but does not identify the oxidised substrate, consuming cell or ATP output (109–112). Respirometry and extracellular-flux assays quantify capacity under assay conditions rather than intact-brain recruitment during cognition (113–115).

High-energy-phosphate measurements likewise separate state from turnover. Conventional ^31^P MRS measures ATP, phosphocreatine and inorganic-phosphate pools, whereas magnetisation transfer provides model-dependent exchange estimates (16, 116, 117). Genetically encoded ATP/ADP and redox sensors add temporal and subcellular resolution but still integrate production, consumption, transport and buffering (11, 15, 118–120). No single readout establishes functional sufficiency; inference should therefore stop at the last measured link.

### 4.4 Mapping measurements to permissible claims

Interpretation should remain closest to the measured domain (**Table 1**). Molecular capacity, static pools, task-evoked proxies, model-derived rates, oxidative or energetic state and neural or cognitive outcomes form an inferential map, not a quality ranking or obligatory biological sequence (16, 36, 64, 80, 95, 103). Cross-level causal claims require temporal alignment, appropriate spatial and cellular resolution, and perturbation or rescue linking the proposed steps (11, 80, 107). A neural or cognitive deficit establishes a functional phenotype, and an unchanged mean does not establish intact adaptation. Inference should stop at the last process, timescale and compartment directly resolved.

## 5 Neurometabolic responses to cognitive and metabolic challenges in diabetes

The four challenge categories are grouped according to the primary experimental input being manipulated, rather than according to an assumed disease mechanism. Cognitive-task challenges increase endogenous neural demand; glucose or insulin challenges alter hormonal or substrate availability; hypoglycaemic or alternative-substrate challenges stress fuel availability and compensatory flexibility; and controlled neural or network challenges impose more spatially or temporally defined circuit activity (**Figure 3**). These categories are pragmatic perturbation classes rather than mutually exclusive biological mechanisms, and some experiments may span more than one category. Importantly, challenge paradigms are not failure phenotypes: any challenge may reveal more than one phenotype, and the same phenotype may be elicited by different challenges. Together, these paradigms resolve selected links extending from vascular recruitment and substrate entry to oxidative flux, energetic state and neural function, but the demand-to-cognition chain remains untested end-to-end in clinically characterised DCI.

**Figure 3:**
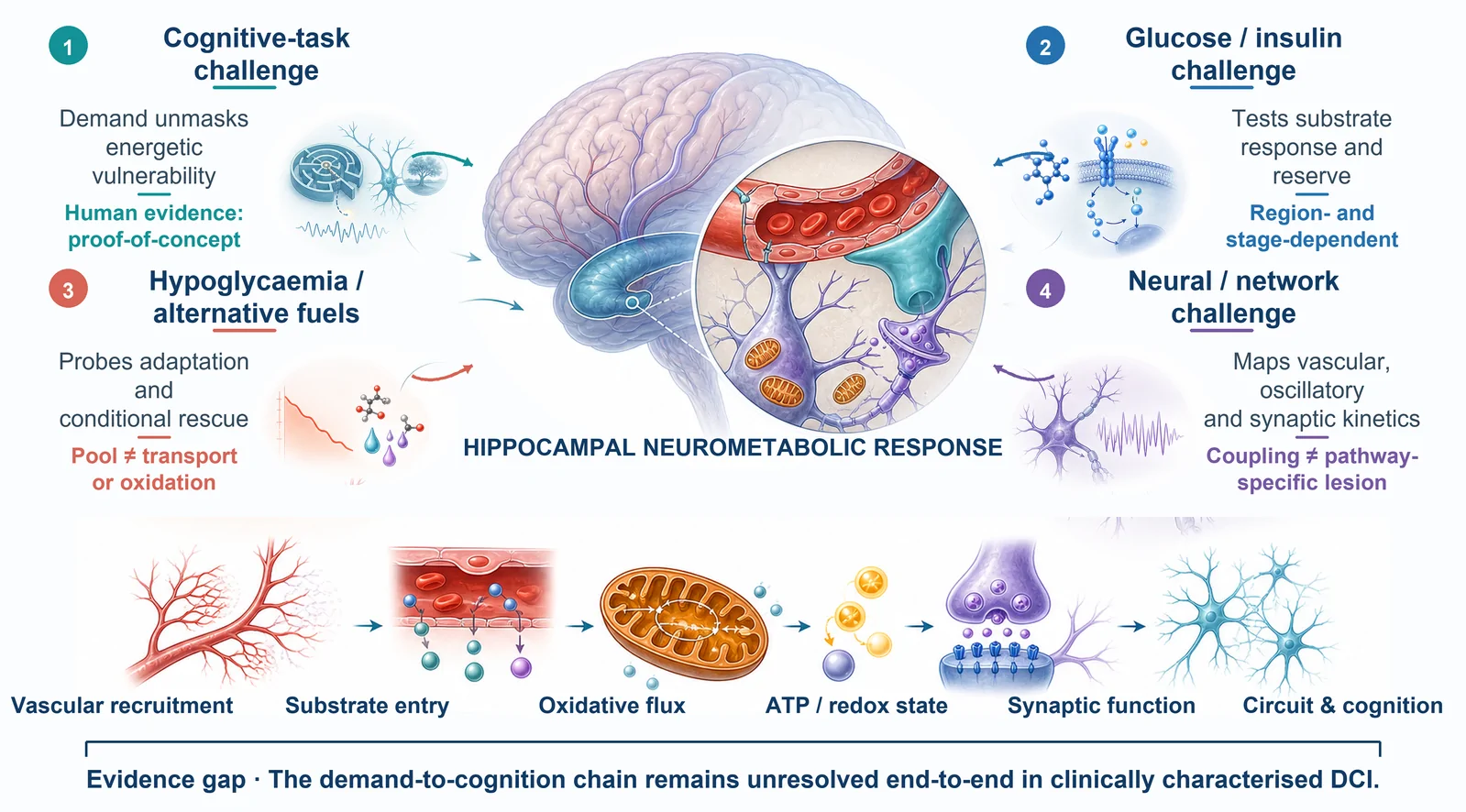
Complementary challenge paradigms for interrogating activity-dependent neurometabolic adaptation in diabetes-associated cognitive impairment.

### 5.1 Cognitive-task challenges: demand-evoked neurometabolic vulnerability

Cognitive testing is a physiological perturbation that raises regional energy demand. In healthy preparations, demanding spatial tasks lower hippocampal extracellular glucose, astrocytic glycogen-derived lactate supports long-term memory, and sustained firing requires ongoing glycolytic and mitochondrial ATP supply (10–12). Contextual learning also increases neuronal glucose uptake and surface GLUT3 recruitment; disrupting these responses lowers event-related glucose and ATP signals and impairs memory acquisition (126, 127). The relevant reference state is therefore the capacity to sustain energetic support as demand rises, rather than transporter or metabolite abundance at rest.

Human studies of metabolism during cognition remain scarce. Under acute lipid loading, sustained testing in healthy adults was accompanied by a lower hippocampal PCr/ATP ratio without poorer overall performance (17). This result identifies a demand-sensitive change in high-energy-phosphate balance, not impaired ATP synthesis: acquisition was not tightly task-locked, no formal lipid-by-task interaction was reported, and the ratio cannot distinguish greater phosphocreatine use from deficient ATP regeneration. More disease-proximal evidence comes from older adults with prediabetes or early type 2 diabetes. During memory encoding, they showed a more diffuse pattern of FDG-derived cerebral glucose metabolism and poorer delayed recall than normoglycaemic controls (123). Because uptake was integrated over a prolonged period and the control group was small, the study supports altered metabolic recruitment but not a temporally resolved failure of glucose flux.

Task-evoked neural phenotypes in established type 2 diabetes are heterogeneous. Memory-task fMRI has shown reduced dorsolateral prefrontal recruitment and incomplete default-mode deactivation despite preserved performance (128). In graded n-back tasks, frontal recruitment weakened progressively as load increased in some cohorts (129). By contrast, newly diagnosed, untreated type 2 diabetes was associated with greater frontoparietal recruitment only at the highest load (130). Acute hyperglycaemia also abolished the normal increment in frontal and anterior cingulate BOLD response from intermediate to high working-memory load in participants with type 2 diabetes, but not in controls (131). Thus, greater recruitment may support performance early in disease, whereas higher demand or an adverse glycaemic state can expose attenuation; there is no universal hypoactivation signature.

Temporal and vascular measurements further qualify these findings. In type 2 diabetes, magnetoencephalography linked poorer glycaemic control to phase-specific abnormalities during working-memory encoding and maintenance (132). In healthy adults, simultaneous FDG-fPET and BOLD fMRI showed that glucose-related and haemodynamic responses can dissociate across networks (35). In diabetes, adjustment for cerebrovascular reactivity changed the spatial pattern of working-memory BOLD associations with HbA1c (101). BOLD abnormalities therefore establish altered task-evoked haemodynamics, but do not locate the limitation in neural activity, vascular delivery, substrate use or oxidative ATP support.

Cognitive demand is therefore an informative neurometabolic stressor, but activity-dependent energy failure is not yet an established mechanism of diabetes-associated cognitive impairment. Disease-proximal metabolic evidence remains temporally coarse, hippocampal high-energy-phosphate evidence comes from acute insulin resistance in healthy volunteers, and chronic type 2 diabetes studies largely measure haemodynamic or electrophysiological responses. No clinically characterised study has combined graded cognitive demand with time-resolved substrate flux or ATP-related measures and a neural or behavioural endpoint.

### 5.2 Glucose and insulin challenges: substrate responsiveness and reserve mobilization

Glucose and insulin challenges test cerebral responsiveness to controlled changes in hormonal or substrate availability. A hyperinsulinaemic-euglycaemic clamp varies insulin while maintaining glycaemia, whereas a hyperglycaemic clamp tests the response to increased circulating glucose. In healthy adults, physiological hyperinsulinaemia produced little change in net whole-brain glucose transport or metabolic rate (133). Marked hyperinsulinaemia likewise did not change estimated cerebral glucose utilisation in an early FDG-PET study of type 2 diabetes (134). These whole-brain null responses do not establish cerebral insulin insensitivity, because central insulin effects may be regional, neural or vascular rather than expressed as a large global change in glucose metabolism.

Later studies identified an altered FDG phenotype in systemic insulin resistance. During paired fasting and clamp experiments, insulin increased FDG-derived cerebral glucose metabolism in impaired glucose tolerance and severe obesity, with little increase in lean controls (24, 25). This obesity-associated response diminished after bariatric surgery as peripheral insulin sensitivity improved (25). Whole-body PET/MRI studies and a large pooled cohort also associated lower peripheral insulin sensitivity with higher cerebral FDG uptake during hyperinsulinaemia (26–29). Several reports analysed related or partially overlapping cohorts and should be viewed as complementary characterisation rather than independent replication. Meta-analysis nevertheless supported a state-dependent reversal, with insulin resistance tending to accompany lower fasting metabolism but higher insulin-stimulated values (30). The direction changed under another endocrine design: after suppression of endogenous insulin, physiological replacement elicited an attenuated global and frontostriatal FDG response in insulin-resistant participants (135). Dynamic PET modelling in obese miniature pigs attributed the higher insulin-stimulated signal mainly to increased inward glucose transfer, without corresponding changes in phosphorylation or outward transport (37). FDG trapping therefore indicates altered delivery, transport and phosphorylation, not downstream oxidation or ATP support.

Hyperglycaemia reveals a complementary disturbance. During controlled exposure, adults with obesity or poorly controlled type 2 diabetes showed a smaller rise in occipital brain glucose concentration than lean controls (97). Repeating the protocol after improved glycaemic management produced a larger cerebral response in the same patients (98). These concentration changes integrate influx, utilisation and redistribution, and the intervention was multifactorial. In younger adults with obesity, [1-^13^C]glucose MRS showed a lower model-derived ratio of blood-to-brain transport capacity to glucose utilisation but comparable estimated tricarboxylic-acid-cycle flux (136). Higher FDG trapping during hyperinsulinaemia and weaker glucose-pool accumulation during hyperglycaemia are therefore compatible: they describe different input-output relationships, neither of which isolates oxidative ATP support.

Central insulin responsiveness can also change without mirroring FDG uptake. Insulin produced smaller changes in stimulus-evoked cortical activity in overweight adults (137). In high-fat-fed rats, it failed to recruit hippocampal microvascular perfusion or Akt phosphorylation, alongside separately measured hippocampus-dependent cognitive impairment (138). Intranasal insulin altered regional perfusion and vasoreactivity in a small crossover study of older adults with type 2 diabetes, but cognitive effects were limited (139). These findings implicate neural and vascular responsiveness without identifying cerebral glucose flux as the mediator. Regional animal studies more directly suggest impaired reserve mobilisation. Intrahippocampal insulin enhanced spatial memory and altered extracellular glucose and lactate in lean rats, whereas diet-induced obesity attenuated both responses in parallel experiments (18). Diet-induced diabetes was also associated with lower hippocampal glycogen and little additional net glycogen change after local insulin (19). In Goto-Kakizaki rats, incorporation of glucose-derived carbon into brain glycogen was impaired despite relatively preserved glycogen content, particularly in the hippocampus (122). Together, these data support reduced regional insulin responsiveness and less readily recruitable stored substrate, but not metabolic mediation of memory failure because behaviour and metabolism were not aligned within one challenge window.

Glucose and insulin challenges therefore reveal a state-dependent reorganisation rather than a single diabetic phenotype: responses may be exaggerated, attenuated or preserved according to stimulus, hormonal background, region and model. Hippocampal stored fuel may be less readily mobilised, but whether this limits oxidative ATP support during cognition remains unknown. No study in clinically characterised diabetes-associated cognitive impairment has aligned a standardised metabolic challenge with pathway-specific flux, energetic state and cognitive performance.

### 5.3 Hypoglycaemic and alternative-substrate challenges: conditional adaptation and functional trade-offs

Acute hypoglycaemia lowers the dominant circulating fuel while activating counter-regulatory, cerebrovascular and neural responses and increasing reliance on endogenous reserves or alternative substrates (140, 141). The phenotype therefore depends on both the current glucose nadir and metabolic history. Recurrent exposure can lower the thresholds for autonomic warning and counter-regulation, so preserved cerebral function may coexist with reduced awareness and greater clinical risk (140). Controlled hypoglycaemia tests adaptive range, but most evidence comes from insulin-treated type 1 diabetes and cannot by itself explain chronic cognitive decline in diabetes-associated cognitive impairment.

Recurrent hypoglycaemia illustrates why response direction alone cannot distinguish adaptation from vulnerability. In rats, it abolished the usual task-associated decline in hippocampal extracellular glucose and improved spatial alternation during euglycaemia, yet accelerated the decline and worsened performance during a later hypoglycaemic episode (142). Compartmental 13C modelling showed a corresponding shift: neuronal glucose use increased at euglycaemia, whereas neuronal pyruvate-dehydrogenase flux and total tricarboxylic-acid-cycle activity fell during subsequent hypoglycaemia (143). In healthy adults, recurrent exposure shifted the thresholds for declining brain glucose uptake and cognition while delaying symptoms and hormonal defence (144). Evidence for a simple increase in transport is weak. Model-derived hippocampal transport-to-utilisation ratios did not differ among type 1 diabetes groups with impaired or normal awareness and healthy controls (145). Three hypoglycaemic exposures likewise produced no detectable change in corresponding prefrontal or hypothalamic kinetics during later hyperglycaemic clamps (146). These findings support a changed operating range, while leaving regional, chronic and substrate-specific adaptation unresolved.

Human studies similarly reveal preservation coupled to redistribution or functional cost. Whole-brain glucose uptake can remain preserved during moderate hypoglycaemia in intensively treated type 1 diabetes despite weakened symptoms and counter-regulation (147). Awareness-dependent global and prefrontal metabolic responses have also been detected with 3-O-methyl-D-glucose PET (148). During working memory, adults with type 1 diabetes maintained performance through greater task-network activation and less default-mode suppression (149). Yet standardised testing at comparable glucose levels found cognitive impairment across type 1 diabetes, insulin-treated type 2 diabetes and controls, largely irrespective of awareness status (150). Recent real-world exposure was also unrelated to the magnitude of cognitive decline during a later clamp (151). Hypoglycaemia nevertheless produced a modest working-memory cost and regional cerebral-blood-flow redistribution in a crossover study (124). Arterial-spin labelling subsequently revealed disrupted vascular oscillatory regulation in type 1 diabetes and weaker regional perfusion recruitment with impaired awareness (152). Preserved performance, uptake or flow therefore need not represent an unchanged cerebral response.

Metabolic measurements also reveal substrate-specific adaptation. The occipital PCr/ATP ratio remained stable during moderate hypoglycaemia but was lower throughout in type 1 diabetes than in controls (153). This ratio does not measure ATP synthesis. Model-derived tricarboxylic-acid-cycle flux was preserved during [1-13C]glucose clamps in healthy adults (154). It was also maintained within type 1 diabetes and exceeded a historical healthy estimate during hypoglycaemia, although the sample was small, incompletely paired and compared with noncontemporaneous controls (155). Acetate transport and oxidation were higher during hypoglycaemia in well-controlled type 1 diabetes (156). Absolute cortical acetate metabolism was also higher with impaired awareness than with normal awareness or in healthy controls (157). This cannot be generalised to other fuels. Frequent hypoglycaemia was associated with a larger calculated brain lactate pool without greater lactate-derived glutamate or glutamine enrichment (9). Exercise-induced hyperlactataemia produced awareness-dependent brain lactate kinetics (158). In recurrently hypoglycaemic rats, enhanced lactate transport contributed little directly to oxidation, yet lactate preserved glucose metabolism, tricarboxylic-acid-cycle activity and neural function (159). Alternative fuels may thus reshape glucose use rather than simply replace it.

Interventions show that alternative fuels can support function when glucose is scarce. Oral medium-chain triglycerides protected several cognitive measures during hypoglycaemia in adults with type 1 diabetes, and beta-hydroxybutyrate sustained synaptic transmission in rat hippocampal slices exposed to low glucose (160). The human study did not trace cerebral fuel use, and the cross-species observations do not form one causal chain. Complementary 2026 work found a greater relative contribution of labelled beta-hydroxybutyrate to cerebral oxidation in type 1 diabetes because total oxidative flux was lower, not because absolute ketone oxidation was higher. In a separate cohort, medium-chain triglycerides improved working memory and regional BOLD activation during hypoglycaemia without altering counter-regulatory hormones (161). The metabolic and functional findings came from different cohorts, and BOLD does not measure ATP production. Brain slices from db/db mice likewise showed lower glucose metabolism but greater acetate and beta-hydroxybutyrate use, while isolated mitochondria had higher oxygen-consumption and ATP-synthesis capacity (162). These ex vivo capacities demonstrate residual flexibility, not restored *in vivo* task energetics.

Responses to low glucose depend on exposure depth and duration, prior hypoglycaemia, awareness status, brain region and the selected endpoint. Preserved oxidative flux can coexist with impaired autonomic warning, and preserved accuracy with broader network recruitment; larger substrate pools or functional rescue do not establish greater endogenous oxidation. No study in clinically characterised diabetes-associated cognitive impairment has combined a cognitive task, controlled glucose restriction, absolute alternative-substrate flux, energetic state and performance. Recurrent hypoglycaemia therefore defines a conditional boundary between compensation and failure rather than an established mechanism of chronic cognitive impairment.

### 5.4 Neural and network challenges: stimulus-evoked neurovascular, oscillatory, and synaptic responses

Controlled neural challenges impose defined inputs and resolve physiological outputs more precisely than whole cognitive tasks. Sensory stimulation, oscillations, sharp-wave ripples and long-term potentiation probe vascular, network or synaptic responsiveness under load. Functional hyperaemia arises from coordinated signalling among neurons, astrocytes, mural cells and endothelium (14, 50). It measures vascular recruitment rather than glucose use or ATP synthesis, and blood flow can diverge from glucose uptake in activated cortex (163). Conversely, abnormal oscillations or potentiation establish circuit dysfunction without identifying the limiting metabolic step.

Non-diabetic experiments define the metabolic relevance of these challenges. Electrical activity rapidly recruits glycolytic and mitochondrial ATP synthesis at presynaptic terminals, and interrupting that recruitment impairs vesicle cycling despite a substantial resting ATP pool (11). Loss of the mitochondrial calcium uniporter reduces hippocampal gamma synchrony, alters sharp-wave ripples and prevents activity-related pyruvate-dehydrogenase dephosphorylation (94). In hippocampal slices, lactate alone preserved lower-demand sharp-wave ripples but disrupted gamma and theta-gamma activity despite increased oxygen consumption (164). Conversely, gamma oscillations remained stable during moderate neuroinflammation through greater mitochondrial ATP production and oxygen use despite a changed tissue lactate-to-glucose ratio (165). Network support therefore depends on demand, substrate mixture and the pathway of ATP supply, not substrate availability alone.

Diabetes studies show heterogeneous stimulus-evoked vascular responses. In streptozotocin-diabetic rats, sensory stimulation produced smaller pial arteriolar dilations and impaired BKCa- and Kir-dependent reactivity; PKC inhibition restored these responses, and later islet transplantation prevented them (166, 167). In young Goto-Kakizaki rats, glutamatergic activation evoked briefer nitric-oxide transients and smaller, shorter hippocampal microvascular responses alongside impaired spatial memory (125). Other abnormalities are mainly temporal. Diabetic mice had largely preserved resting perfusion and overall response magnitude but delayed peak recruitment; glyoxalase-1 overexpression normalised the delay and limited visuospatial impairment (20). Luseogliflozin improved functional hyperaemia, vascular integrity and cognition in aged diabetic rats, although systemic glucose-lowering and vascular effects preclude assignment to one neural-metabolic step (168). Akita mice instead retained cortical neurovascular coupling across disease progression (169). Their dilation became more prolonged with age rather than uniformly smaller. Model, region, age, anaesthesia, stimulus and response metric therefore shape the apparent phenotype, and none of these studies measured challenge-locked metabolic flux or ATP turnover.

Oscillatory challenges reveal circuit abnormalities that resting measurements miss. Kainate-evoked hippocampal gamma activity in Goto-Kakizaki rats shifted towards faster components and became less variable, with altered parvalbumin-interneuron markers (170). In type 2 diabetic mice, oscillations slowed, theta-gamma coupling and cortico-hippocampal synchrony weakened, and sharp-wave ripples became longer with greater ripple-associated gamma power (171). These patterns indicate temporal reorganisation but do not identify substrate use or energetic state. In streptozotocin-diabetic mice, exogenous L-lactate improved long-term object recognition and ex vivo sharp-wave-ripple amplitude in separate experiments (172). This rescue demonstrates substrate-responsive capacity, not endogenous lactate deficiency, oxidation or metabolic mediation of memory recovery. Across network states, lactate is not uniformly sufficient (164).

Long-term potentiation links metabolic support to durable synaptic change, but its impairment is mechanistically non-specific. In diabetic rat hippocampal slices, pyruvate or insulin restored potentiation, whereas additional glucose did not (173). Acute GLUT4 blockade disrupted CA3-CA1 transmission, synchronisation and potentiation, with several deficits rescued by D-β-hydroxybutyrate (174). These interventions show substrate flexibility but do not reproduce chronic diabetes. Mitochondrial rescue provides a stronger disease link. In db/db mice, GSK3β-Drp1-dependent fragmentation, impaired complex I activity and lower ATP production accompanied defective potentiation; pathway modulation restored plasticity (175). In streptozotocin-diabetic mice, cyclophilin-D deletion protected ATP-synthase function, respiration, potentiation and cognition (176). Because metabolic assays and synaptic challenges were not aligned temporally, neither study identifies the energetic step failing during potentiation. Hippocampal insulin-receptor knockdown also impaired potentiation and learning without systemic dysglycaemia, showing that receptor signalling can produce a similar endpoint (177). High-fat feeding was associated with lower brain glucose uptake, altered neuronal and astrocytic carbon metabolism and weaker potentiation (178). Lipoic acid improved these domains, but measurements were neither synchronous nor pathway-specific. Presynaptic pyruvate transport, astrocytic glycogen and lactate transfer, neuronal translation and activity-triggered GLUT4 insertion can each support synaptic function (12, 44, 47, 72, 76). Their roles during a diabetes-relevant challenge remain unresolved.

Diabetes can alter the amplitude, timing or duration of evoked vascular responses and reorganise oscillatory and synaptic activity under load, but preserved coupling and successful substrate rescue show that residual capacity can coexist with vulnerability. Reduced gamma activity, abnormal sharp-wave ripples and impaired potentiation should therefore be treated as challenge-sensitive functional endpoints, not metabolic diagnoses. The literature has not yet located the first limiting energetic step connecting these abnormalities to diabetes-associated cognitive impairment.

## 6 An integrated framework for activity-dependent neurometabolic failure in DCI

Activity-dependent neurometabolic failure concerns inadequate matching of metabolic support to neural demand within a defined challenge rather than a uniform reduction in brain metabolism. Established mechanisms and the proposed phenotypes answer different questions. Neurovascular dysfunction, brain insulin resistance and mitochondrial impairment identify candidate lesion classes, whereas the four phenotypes describe how a failure is expressed at a measured support node during a defined challenge and which observations could distinguish it from competing explanations (**Figure 4**). The phenotypes are non-exclusive response descriptors rather than mutually exclusive causal diagnoses. A downstream utilisation defect may secondarily alter upstream recruitment through feedback, while an upstream delivery defect may propagate to downstream energetic failure. A single lesion may therefore produce different phenotypes across workload, disease stage or compensatory state, while the same phenotype may arise from different lesions. Phenotype assignment should remain tied to the response dimension and node actually measured rather than inferred across unmeasured links. Neurometabolic reserve denotes the additional support recruitable above baseline. Reserve exhaustion requires repeated within-person evidence that this range progressively contracts under comparable demand and physiological conditions and that the contraction precedes and predicts neural or cognitive failure.

**Figure 4:**
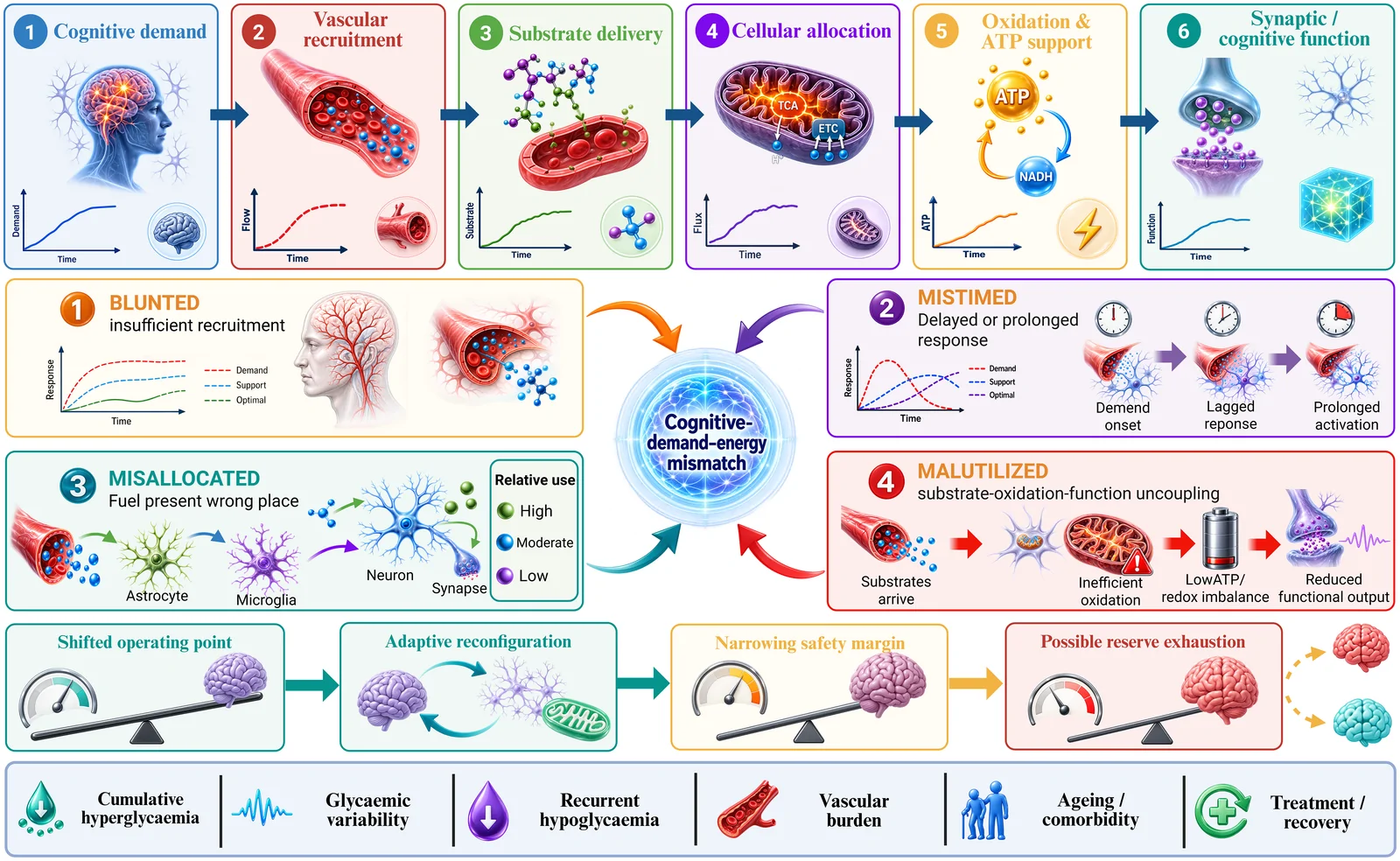
An integrated framework for activity-dependent neurometabolic failure in diabetes-associated cognitive impairment. The four central phenotypes describe candidate abnormalities within a defined challenge, whereas the lower sequence schematically illustrates possible changes in recruitable capacity with metabolic history. Neurometabolic reserve denotes the additional support recruitable above baseline. In this framework, reserve exhaustion requires repeated within-person evidence of a progressively contracting recruitable response range under comparable demand and physiological conditions, with the contraction preceding and predicting neural or cognitive failure. A single attenuated response does not establish reserve exhaustion. The lower sequence represents a proposed, non-obligatory trajectory rather than fixed disease stages; stabilisation or partial recovery may occur.

The four proposed phenotypes have different evidential bases and should not be interpreted as equally established mechanisms of DCI. **Table 2** summarises the study-level findings, their evidential roles and the remaining inferential boundaries. Challenge-based observations support abnormalities at selected vascular and substrate-pool readouts, whereas cellular misallocation and downstream utilisation failure rely more heavily on preclinical modelling or perturbation studies. Support for an individual component does not establish the complete demand-to-function pathway in clinically characterised DCI.

**Table 2:** Evidence proximity to clinically characterised DCI, study-level support and inferential boundaries of the four proposed neurometabolic failure phenotypes.

Reference	Population/model and clinical relevance	Challenge and readout	Reported finding	Evidence role and directness	Inferential boundary
Blunted recruitment Proposed signature. A smaller challenge-evoked response or input–output slope at a specified support node, after accounting for baseline state, systemic input and neural engagement. Evidence status. Challenge-based support at selected vascular and substrate-pool readouts; deficient cognitive-task-evoked oxidative or ATP recruitment remains unconfirmed.					
Nealon et al. (100)	Adults with T2D and matched controls. Other human diabetes evidence. *Cognition assessed; clinical DCI is not established by lower group-average performance.*	Cognitive tasks; transcranial Doppler measurement of middle-cerebral-artery flow velocity.	Resting flow velocity was similar, but task-evoked cerebrovascular responses were smaller and cognitive performance was poorer in T2D.	Human task-evoked vascular observation.	Arterial velocity is not regional substrate delivery. Without matched neural drive, the finding does not identify a metabolic bottleneck or metabolic mediation.
Hwang et al. (97)	Adults with obesity or poorly controlled T2D; lean controls. Mixed human evidence. *T2D: other human diabetes evidence; obesity: other human evidence. Relevance is subgroup-specific.*	Matched hyperglycaemic clamp; occipital brain-glucose concentrations measured by MRS.	Brain-glucose concentration rose less in obesity and T2D during matched hyperglycaemia.	Human dynamic substrate-pool observation.	No cognitive task. A smaller concentration increment does not distinguish reduced input from increased use, or establish deficient oxidation or ATP production.
Sanchez-Rangel et al. (98)	Eight adults with T2D; within-person pre–post study. Other human diabetes evidence. *No cognitive endpoint; clinical DCI applicability remains undetermined.*	Hyperglycaemic clamp and brain-glucose MRS before and after 12 weeks of intensified glycaemic management.	The brain-glucose concentration response increased after improved glycaemic control.	Human reversibility evidence for a pool response.	Small, uncontrolled, multifactorial intervention; no cognitive endpoint or pathway-specific flux measurement.
Emmanuel et al. (17)	Healthy adults with acute lipid-induced insulin resistance. Other human evidence. *Acute experimental proof of principle, not a clinical DCI cohort.*	Sustained cognitive testing; hippocampal PCr/ATP measured by ³¹P MRS.	PCr/ATP fell during cognitive testing with lipid infusion, but not in the no-lipid condition; overall performance did not deteriorate.	Human proof of principle for demand-associated energetic vulnerability; not phenotype-specific validation.	Acute, small, non-DCI model; no formal lipid-by-task interaction reported. PCr/ATP does not distinguish increased expenditure or buffering from deficient ATP synthesis.
McNay et al. (18)	High-fat-fed, insulin-resistant rats and chow-fed controls. Preclinical diabetes/insulin-resistance evidence.	Local hippocampal insulin; memory testing and extracellular glucose/lactate microdialysis in parallel experiments.	Both mnemonic and extracellular glucose/lactate responses to hippocampal insulin were attenuated by high-fat feeding.	Parallel behavioural and metabolic evidence of impaired insulin responsiveness.	Exogenous insulin and separate experiments do not establish metabolic mediation of memory. Microdialysis measures concentration, not carbon flux.
McNay (19)	Diet-induced diabetic rats and controls. Preclinical diabetes/insulin-resistance evidence.	Local hippocampal insulin; one post-challenge glycogen-pool endpoint.	Hippocampal glycogen was lower; no additional net decrease after insulin was detected in diabetic rats.	Animal substrate-pool response to insulin.	No cognitive endpoint. A single net pool change cannot distinguish glycogen synthesis, breakdown or a baseline floor effect.
Mistimed recruitment Proposed signature. An altered onset, peak, duration or recovery that is misaligned with the neural event requiring support; a normal peak or integral does not exclude such misalignment. Evidence status. Human and animal evidence for altered haemodynamic timing; extension to cellular substrate flux, oxidation or ATP timing remains inferential.					
Duarte et al. (21)	Adults with T2D and matched controls. Other human diabetes evidence. *Performance-matched task; a clinical DCI phenotype is not established by this comparison.*	Performance-matched visual discrimination; event-related fMRI haemodynamic response profiles.	Haemodynamic response profiles differed in T2D despite matched task performance.	Human task-evoked haemodynamic observation.	BOLD combines neural and vascular influences; it does not isolate substrate metabolism or establish delayed cellular energy supply.
Duarte et al. (22)	Expanded T2D/control cohort, including participants from (21). Other human diabetes evidence. *Overlapping cohort; clinical DCI applicability remains undetermined.*	Event-related fMRI; response rise, peak, amplitude and post-stimulus recovery across regions.	T2D was associated with slower response evolution, later peaks and altered amplitude/recovery.	Expanded human analysis of haemodynamic kinetics.	Not an independent replication of (21). Metabolic response timing and its relation to independent neural timing were not resolved.
Berends et al. (20)	STZ-diabetic mice with or without Glo1 overexpression. Preclinical diabetes/insulin-resistance evidence.	Whisker-evoked cortical blood-flow kinetics; separate later visuospatial testing.	Recruitment was delayed despite preserved peak/integrated flow; Glo1 overexpression shortened the delay and limited later learning impairment.	Animal vascular perturbation evidence; also limits universal blunting.	Systemic overexpression and separate vascular/behavioural measurements do not demonstrate task-aligned metabolic mediation.
Gonçalves et al. (125)	Young Goto–Kakizaki rats and controls. Preclinical diabetes/insulin-resistance evidence.	Local glutamatergic stimulation; hippocampal nitric-oxide/blood-flow kinetics; separate spatial-memory testing.	Nitric-oxide transients and microvascular responses were smaller and shorter, alongside poorer spatial memory.	Animal vascular amplitude–duration evidence; relevant to blunting and mistiming.	Stimulation and memory testing were not one task-locked metabolic experiment; nitric-oxide/flow kinetics do not measure substrate or ATP flux.
Chiang et al. (169)	Akita mice followed longitudinally. Preclinical diabetes/insulin-resistance evidence.	Two-photon imaging of stimulus-evoked cortical neurovascular coupling.	Peak coupling remained preserved across disease progression; older diabetic mice showed more prolonged dilation.	Temporal heterogeneity and evidence against universal vascular blunting.	Prolonged dilation alone is not proof of harmful mistiming. No cognitive endpoint or metabolic-flux measurement.
Ugidos et al. (179)	STZ-treated mice exposed to recurrent non-severe hypoglycaemia. Preclinical diabetes/insulin-resistance evidence.	Awake two-photon imaging of stimulus-evoked penetrating-arteriole dilation.	Recurrent hypoglycaemia produced a transient delay without reducing peak or integrated dilation; sustained hyperglycaemia alone did not reproduce the delay.	Exposure-dependent vascular kinetic evidence.	A sensory vascular response, not a cognitive-task metabolic response; does not identify delayed substrate entry, oxidation or ATP regeneration.
Candidate substrate misallocation Proposed signature. An input-controlled shift in substrate delivery, retention or fate away from the task-relevant cell or compartment that is associated with inadequate energetic or functional support. Redistribution without demonstrated functional cost remains descriptive. Evidence status. Predominantly preclinical, model-derived and cell-associated mechanistic support; demand-specific pathological allocation remains a hypothesis.					
Girault et al. (180)	Goto–Kakizaki rats and controls. Preclinical diabetes/insulin-resistance evidence.	Dynamic *in vivo* [1, 6-¹³C]glucose MRS and neuronal–astrocytic compartmental modelling.	Models estimated lower neuronal TCA activity, higher astrocytic TCA activity and reduced glutamate–glutamine cycling.	Model-derived compartmental metabolic evidence.	Compartmental rates depend on precursor input, exchange and model structure; no task-locked demonstration of carbon diversion from active neurons.
Zheng et al. (181)	db/db mice with cognitive decline and controls. Preclinical diabetes/insulin-resistance evidence.	Acetate- and lactate-derived ¹³C labelling in brain metabolites.	Acetate-derived labelling decreased whereas lactate-derived labelling increased in selected metabolites.	Substrate-dependent metabolic reorganisation.	Labelling depends on input, dilution and pool size. It does not directly establish cell-to-cell transfer or pathological allocation during cognition.
Chen et al. (182)	db/db mice with endothelial-enriched Hif1a knockdown. Preclinical diabetes/insulin-resistance evidence. *Animal intervention component.*	Endothelial glycolytic features, bulk hippocampal lactate, memory and neurogenesis.	Knockdown reduced glycolytic features and lactate accumulation while improving memory and neurogenesis, without correcting systemic glycaemia.	Endothelial-associated perturbation evidence.	Incomplete lineage restriction, bulk lactate and HIF-1α pleiotropy limit source/fate attribution. Neuronal glucose deprivation was not demonstrated.
Li et al. (183)	db/db mice with hippocampal AAV-shRNA Khk knockdown; complementary microglial experiments. Preclinical diabetes/insulin-resistance evidence.	Fructose metabolism; mitochondrial, synaptic, electrophysiological and cognitive readouts.	Microglia-associated KHK metabolism was implicated; hippocampal knockdown improved mitochondrial, synaptic and cognitive phenotypes.	Hippocampal perturbation with microglial mechanistic support.	The *in vivo* intervention was not microglia-specific conditional deletion. It did not show task-locked substrate capture at the expense of neurons.
Malutilization Proposed signature. Relatively preserved substrate entry or availability accompanied by disproportionately impaired downstream oxidation, energetic support or function during the same challenge. Evidence status. Preclinical intervention-based support for mitochondrial constraints, with indirect human pool–use evidence; the complete within-challenge phenotype remains unconfirmed.					
De Feyter et al. (9)	Adults with T1D and controls during hypoglycaemia. Other human diabetes evidence. *Acute hypoglycaemic adaptation; a chronic DCI phenotype is not established.*	[3-¹³C]lactate infusion and brain MRS; calculated lactate pool and downstream fractional enrichment.	The calculated brain-lactate pool was larger in T1D, without greater lactate-derived glutamate/glutamine enrichment.	Human substrate-pool–use dissociation.	No cognitive challenge. Similar fractional enrichment does not establish reduced absolute lactate oxidation or an ATP deficit.
Yan et al. (176)	STZ-diabetic mice with or without cyclophilin-D deficiency. Preclinical diabetes/insulin-resistance evidence.	Mitochondrial permeability, ATP-synthase activity, respiration, oxidative stress, LTP and memory.	Cyclophilin-D deficiency protected ATP-synthase activity and respiration and alleviated plasticity and memory deficits.	Genetic perturbation linking mitochondrial and functional outcomes.	No aligned measurement of preserved substrate entry and deficient energetic support during cognition; separate readouts do not establish metabolic mediation.
Huang et al. (175)	db/db mice and high-glucose neuronal models. Preclinical diabetes/insulin-resistance evidence.	Drp1/GSK3β modulation; mitochondrial dynamics, complex-I activity, ATP-related measures and LTP.	Pathway modulation mitigated mitochondrial fragmentation, complex-I/ATP abnormalities and impaired LTP.	Perturbation-based downstream mitochondrial support.	No paired substrate-entry and task-evoked oxidation measurement; LTP improvement does not identify the energetic mediator.
Chandrasekaran et al. (184)	STZ-diabetic rats receiving NMN from early diabetes. Preclinical diabetes/insulin-resistance evidence.	Hippocampal metabolites/NAD^+^; isolated-mitochondrial respiration and spare capacity; CA1 neurons and memory.	Glucose accumulation coexisted with lower NAD^+^ and respiration; NMN preserved respiration/spare capacity, CA1 neurons and memory without correcting systemic glycaemia.	Preventive intervention linking respiratory and functional protection.	Respiratory assays were ex vivo, not task-evoked flux measurements. Early treatment does not demonstrate reversal of established cognitive impairment.
Malutilization					
Ruegsegger et al. (185)	High-fat-fed mice receiving exercise or metformin. Preclinical diabetes/insulin-resistance evidence.	ATP-production capacity in isolated cerebral mitochondria and oxidative-enzyme activity.	High-fat feeding reduced mitochondrial ATP-production capacity and oxidative-enzyme activity; interventions improved these measures.	Mitochondrial-capacity and reversibility evidence.	No cognitive endpoint or *in vivo* demand-evoked metabolism. Assay capacity is not equivalent to ATP support at engaged synapses.
Ruegsegger et al. (186)	STZ-diabetic mice after insulin withdrawal; separate non-diabetic mice given intranasal insulin. Mixed preclinical evidence. *Diabetes-related withdrawal experiment; separate non-diabetic reference experiment.*	Cerebral mitochondrial ATP production, oxidative enzymes and monocarboxylate-related measures.	Withdrawal reduced ATP production. Intranasal insulin increased ATP production per tissue mass, but not per mitochondrial protein, in non-diabetic mice.	Separate insulin-deficiency and insulin-administration experiments.	Not a diabetic intranasal-insulin rescue experiment. Normalisation matters; no cognition or task-locked substrate-to-ATP measurement.
Zhao et al. (187)	Diabetic mice treated with FGF21. Preclinical diabetes/insulin-resistance evidence.	Hippocampal lactate, MCT2, LDH-B activity, NAD^+^, ATP and memory.	FGF21 lowered lactate and increased MCT2, LDH-B activity, NAD^+^ and ATP, alongside improved memory.	Intervention linking metabolic markers with functional improvement.	Expression, enzyme activity and pool measures do not quantify lactate uptake or oxidation flux; systemic and signalling effects remain possible.
Mason et al. (156)	Adults with well-controlled T1D and controls. Other human diabetes evidence. *Acute hypoglycaemic challenge; a chronic DCI phenotype is not established.*	Hypoglycaemic clamp; [2-¹³C]acetate MRS and metabolic modelling.	Brain acetate concentration and model-derived acetate utilisation relative to TCA cycling were higher in T1D.	Substrate-specific limiting evidence against universal malutilization.	Model-dependent and challenge-specific; no cognitive task. Enhanced use of one alternative fuel does not establish intact overall energetic support.
Andersen et al. (162)	Brain slices and isolated mitochondria from db/db mice. Preclinical diabetes/insulin-resistance evidence.	Glucose, acetate and β-hydroxybutyrate tracing; mitochondrial respiration and ATP-production assays.	Lower glucose-derived labelling coexisted with increased use of selected alternative fuels and higher mitochondrial respiratory/ATP-producing capacity.	Preclinical limiting evidence for residual metabolic flexibility.	Ex vivo substrates and capacity assays do not establish adequate *in vivo* ATP delivery during cognition or refute a conditional utilisation deficit.

Clinical relevance. Source labels apply to the population or experimental component supporting each listed finding, not every component of a report. Direct clinical DCI evidence requires explicitly characterised cognitive impairment in participants with diabetes; diabetes status, lower group-average performance or acute challenge effects alone do not establish it. Where cognitive status is insufficiently characterised, clinical DCI applicability remains undetermined; normal cognition is not assumed.

Source categories. Other human diabetes evidence concerns T1D or T2D without an established clinical DCI phenotype for the reported comparison. Other human evidence concerns non-diabetic metabolic-risk or experimentally challenged participants. Preclinical diabetes/insulin-resistance evidence comprises the specified animal, cellular or ex vivo models; separate non-diabetic reference experiments remain distinct. Mixed sources retain their component-specific relevance. These categories are not quality or certainty grades.

Interpretation and scope. Clinical relevance is assessed separately from measurement directness and mechanistic support. Proposed signatures are experimental criteria for testing mechanistic hypotheses, not clinical diagnostic criteria or findings established by every entry. The four phenotypes are non-exclusive candidates, not validated DCI subtypes. These phenotypes describe responses within a challenge; reserve exhaustion requires longitudinal evidence of a progressively contracting recruitable response range and cannot be inferred from a single attenuated response. The matrix includes 25 distinct reports (21); and (22) have overlapping participants. No included study resolves the complete demand–substrate-fate–energetics–function pathway in clinically characterised DCI. Reference numbers follow the main manuscript bibliography.

AAV, adeno-associated virus; ATP, adenosine triphosphate; BOLD, blood oxygen level-dependent; DCI, diabetes-associated cognitive impairment; Drp1, dynamin-related protein 1; FGF21, fibroblast growth factor 21; fMRI, functional magnetic resonance imaging; Glo1, glyoxalase 1; GSK3β, glycogen synthase kinase 3β; HIF-1α, hypoxia-inducible factor 1α; KHK, ketohexokinase; LDH-B, lactate dehydrogenase B; LTP, long-term potentiation; MCT2, monocarboxylate transporter 2; MRS, magnetic resonance spectroscopy; NAD^+^, nicotinamide adenine dinucleotide; NMN, nicotinamide mononucleotide; PCr, phosphocreatine; shRNA, short hairpin RNA; STZ, streptozotocin; T1D and T2D, type 1 and type 2 diabetes; TCA, tricarboxylic acid cycle.

### 6.1 Blunted recruitment: insufficient scaling of metabolic support with demand

Blunted recruitment is a defect of response gain relative to effective neural demand, not a synonym for resting cerebral hypometabolism or low task activation. In cognitive challenges, it denotes attenuated scaling of a prespecified energy-support response with effective demand, relative to a task- and readout-specific reference. This interpretation requires accounting for baseline state, imposed load, achieved performance, task-relevant neural engagement where available and systemic input. Without adequate characterisation of neural engagement, a smaller response remains descriptive and cannot distinguish deficient metabolic adaptation from reduced effective demand. The expected direction must also be specified for each readout, because a smaller signal is not uniformly pathological. A normal baseline may therefore conceal restricted dynamic reserve, whereas a low resting value may coexist with preserved scaling. In healthy preparations, hippocampal extracellular glucose responses increase with spatial-task demand, and active synaptic terminals recruit glycolysis and mitochondrial ATP synthesis to maintain presynaptic ATP (10, 11). Blunting should consequently be localised to vascular recruitment, substrate entry or mobilisation, oxidative metabolism, or energetic support rather than assigned to “brain metabolism” as a whole.

Human evidence is suggestive but incomplete. In healthy adults exposed to acute lipid loading, sustained cognitive testing was associated with a lower hippocampal PCr/ATP ratio, whereas a small resting cohort showed no comparable change (17). This study demonstrates that metabolic strain can emerge under demand, but it does not model chronic DCI or measure ATP-synthesis or glucose flux. In prediabetes or early type 2 diabetes, memory encoding elicited a more spatially diffuse FDG-derived metabolic response rather than a uniform decrement (123). Established type 2 diabetes has also been associated with weaker cognitive-task-evoked cerebrovascular responsiveness despite similar resting flow velocity (100)and less expansion of frontal recruitment with increasing working-memory load (129). Memory-task fMRI shows reduced dorsolateral prefrontal recruitment with incomplete default-mode deactivation (128).Acute hyperglycaemia can also abolish the normal BOLD increment from intermediate to high working-memory load (131). These vascular and neural findings are consistent with reduced recruitment but are not direct measurements of metabolic flux.

Non-cognitive metabolic challenges reveal a similar loss of responsiveness. Hyperinsulinaemia produces a smaller cerebrocortical response in overweight adults (137), while controlled hyperglycaemia elicits a smaller rise in occipital brain glucose in obesity and poorly controlled type 2 diabetes (97). That response increases after improved glycaemic management (98), indicating that recruitment can remain modifiable rather than irreversibly lost.

Animal studies provide mechanistic bridges at the level of stored fuel and insulin-responsive substrate handling. Intrahippocampal insulin improves memory and changes extracellular glucose and lactate in lean rats, whereas high-fat feeding attenuates both behavioural and metabolic responses (18). In diet-induced diabetes, basal hippocampal glycogen is reduced and local insulin no longer produces the net glycogen decrease observed in controls (19). Because this is a single post-challenge endpoint, altered synthesis, breakdown, and a floor effect cannot be distinguished. Complementary 13C-MRS in Goto-Kakizaki rats showed slower incorporation of glucose-derived carbon into glycogen despite relatively preserved glycogen abundance, together with lower model-derived glucose-transport capacity (122). These findings suggest altered glycogen turnover and insulin-responsive substrate mobilisation, but do not establish reserve exhaustion or trace glycogen-derived carbon into neuronal oxidation during learning.

The delivery arm can also be attenuated. High-fat feeding prevents insulin-mediated recruitment of hippocampal microvascular perfusion and vascular Akt signalling, alongside cognitive impairment assessed separately (138). Sensory stimulation evokes smaller pial arteriolar dilations in streptozotocin-diabetic rats through a PKC-sensitive vascular mechanism (166). Likewise, hippocampal glutamate challenge in young Goto–Kakizaki rats produces briefer nitric-oxide transients and smaller, shorter blood-flow responses in the same small cohort with poorer spatial-memory performance (125). These experiments establish activity-evoked vascular abnormalities, but not reduced glucose or oxygen delivery in the absence of matched neural and substrate measurements.

Blunting is nevertheless not universal. Insulin-stimulated cerebral FDG uptake may increase in systemic insulin resistance (27), and newly diagnosed type 2 diabetes can show greater frontoparietal recruitment at high working-memory load (130). In streptozotocin-diabetic mice, peak and integrated sensory-evoked blood-flow responses can remain near normal despite delayed recruitment (20). Longitudinal imaging in Akita mice likewise found preserved cortical neurovascular coupling across disease progression (169). Such increases or preservation should not automatically be labelled compensation. Response gain varies with disease stage, region, challenge intensity, hormonal state, and the measured step, and adequate upstream recruitment does not guarantee downstream energy support.

Because blood flow and glucose uptake can dissociate (163) and neurovascular coupling resolves only the vascular response (14), blunted recruitment should be assigned to a measured node. Challenge-based abnormalities support selected vascular and substrate-pool phenotypes, whereas deficient oxidative or ATP recruitment during cognition and its causal contribution to clinically characterised DCI remain unresolved (**Table 2**).

### 6.2 Mistimed recruitment: temporal misalignment between neural demand and metabolic support

Neurometabolic adequacy depends on timing as well as magnitude. Mistimed recruitment denotes a support response that is misaligned with the neural event generating energetic demand. It may begin too late, terminate before activity subsides, or persist into recovery despite reaching a normal peak. Functional hyperaemia itself emerges from interactions among neurons, astrocytes, mural cells, and endothelium (50). At active synapses, neuronal glycolysis and local ATP synthesis are recruited rapidly enough to sustain neurotransmission (11, 15). Haemodynamic signals, however, integrate blood flow, blood volume, and oxygenation over distinct kinetics (69). Timing abnormalities must therefore be assigned to the measured step rather than described generically as delayed energy delivery.

The clearest clinical evidence in type 2 diabetes concerns haemodynamic kinetics. During event-related visual discrimination, participants with diabetes showed an altered haemodynamic response despite performance matched to controls (21). A subsequent analysis of the expanded cohort identified a slower rise, later peak, and abnormal post-stimulus recovery, together with amplitude changes (22). These reports are progressive analyses rather than independent replications. Transcranial Doppler studies provide weaker corroboration: visually evoked posterior cerebral artery responses were slower in hypertension and more abnormal when diabetes was also present (188). However, hypertension, the absence of a non-hypertensive diabetic group, and arterial velocity as the outcome prevent attribution to diabetes alone. None of these studies measured substrate flux, oxygen consumption, or high-energy-phosphate turnover.

Animal models more directly show that preserved magnitude can conceal altered kinetics. In streptozotocin-diabetic mice, resting cortical perfusion and peak and integrated whisker-evoked responses remained near normal, but the rise to half-maximum and maximum flow was delayed (20). Glyoxalase 1 overexpression shortened this delay and improved later spatial learning, although systemic intervention and separate vascular and behavioural measurements preclude mediation claims. Awake two-photon imaging subsequently showed that sustained hyperglycaemia could leave penetrating-arteriole kinetics intact, whereas recurrent non-severe hypoglycaemia produced a transient delay without reducing peak or integrated dilation (179). Other models show distinct distortions. Goto–Kakizaki rats generate briefer hippocampal nitric-oxide and blood-flow responses after glutamate challenge (125). Older Akita mice instead show prolonged arteriolar dilation despite preserved peak coupling (169). Mistiming is therefore conditional and is not synonymous with delayed onset. The diversity of trajectories is central to the phenotype. Latency, duration, and recovery are separable response dimensions, and each may interact differently with a brief cognitive operation.

Such distortions may matter because encoding, maintenance, retrieval, and consolidation impose energy costs over different time windows. Diabetic mice show slower hippocampal rhythms, impaired theta–gamma coordination, and prolonged sharp-wave ripples (171), together with abnormal delay-period hippocampal–cortical coupling during working memory (189). These findings establish altered neural timing, not metabolic phase delay. Recurrent hypoglycaemia also changes task-associated hippocampal extracellular glucose trajectories, with an adaptation that benefits performance during euglycaemia but becomes detrimental during acute hypoglycaemia (142). In adults with type 1 diabetes, acute hypoglycaemia slows working-memory performance while redistributing task-related regional blood flow (124). The functional effect of a dynamic response thus depends on both its timing and the prevailing substrate environment. A response adequate during sustained activation may still fail during rapid encoding, whereas prolonged support may be costly or interfere with recovery.

Potential delays may occur in neural-to-vascular signalling, substrate entry or downstream metabolism, but the measured signal localises only the observed step (14, 36, 63, 190, 191). A delayed BOLD or flow response therefore establishes altered haemodynamic recruitment, not delayed transport, oxidation or ATP regeneration. Current evidence supports mistimed recruitment as an emerging haemodynamic phenotype in diabetes. It does not yet establish delayed glucose or lactate flux, oxidation or ATP regeneration during cognition in clinically characterised DCI (**Table 2**). Testing requires task-locked measurements, flexible response models and control of systemic physiology (23, 192).

### 6.3 Substrate redistribution and candidate misallocation: relevance to task-dependent support

Metabolic support may be adequate in magnitude and timing yet fail to reach the compartment carrying the energetic cost. Misallocation denotes a mismatch between where carbon is delivered, retained or transformed and where neural computation occurs. It differs from global substrate scarcity and from inefficient oxidation after delivery. Normal brain metabolism already uses parallel, context-dependent routes. Sensory activation can preferentially increase neuronal glucose uptake (71), whereas hippocampal memory formation can depend on astrocytic glycogenolysis and monocarboxylate transport (12). Cell-resolved imaging has identified an astrocyte-to-neuron lactate gradient (77), and behavioural studies suggest that glucose and monocarboxylate dependence varies across regions and memory phases (47, 126). Neuronal activity can also recruit astrocytic glucose metabolism through adenosine signalling (193). Diabetes may therefore reweight several coexisting routes rather than abolish a single canonical shuttle. A claim of misallocation must specify the substrate, source, recipient, anatomical scale and demand window.

Metabolic redistribution or compartmental reorganisation alone should not be termed misallocation. In this framework, misallocation requires evidence that substrate remains available at an aggregate level but that task-relevant support to the cell, subcellular compartment or biochemical fate bearing the computational load is reduced. Evidence of where the displaced carbon is redirected would strengthen this inference. Without demonstrated compromise of task-relevant energetic or functional support, the more appropriate description is redistribution or metabolic reorganisation.

Available disease evidence is most consistent with reciprocal compartmental remodelling, not literal competition for a fixed glucose pool. Serial *in vivo* [1, 6-^13^C]glucose labelling in Goto–Kakizaki rats yielded model-based estimates of reduced neuronal tricarboxylic-acid-cycle activity, increased astrocytic activity and more strongly depressed glutamate–glutamine cycling (180). In db/db mice, acetate-derived labelling of TCA- and neurotransmitter-related metabolites decreased, whereas lactate-derived labelling increased (181). Ex vivo db/db brain slices also showed less glucose-derived labelling but greater hippocampal use of acetate and beta-hydroxybutyrate (162). Preserved glycogen abundance can similarly coexist with slower incorporation of glucose-derived carbon (122). Hyperpolarised pyruvate studies further indicate stage- and region-dependent pyruvate-to-lactate label exchange (194, 195). These signals do not directly measure competitive, cell-specific carbon flow. Their interpretation depends on tracer delivery, precursor enrichment, pool size, exchange kinetics and model structure.

The brain endothelium is a plausible redistribution site because it controls substrate entry while consuming glucose. Endothelial HIF-1α supports GLUT1 expression and cerebral glucose uptake (196), while endothelial MCT1-dependent transport contributes to hippocampal lactate homeostasis (197). In db/db mice, endothelial-enriched Hif1a knockdown reduced glycolytic features and hippocampal lactate accumulation while improving memory and neurogenesis without correcting systemic glycaemia (182). This supports a pathogenic contribution of endothelial-associated lactate handling, but not neuronal glucose deprivation. The intervention was not absolutely lineage restricted, lactate remained a bulk-tissue pool, and HIF-1α has pleiotropic vascular and inflammatory actions.

Microglia provide another candidate route for pathological redistribution. Suppressing microglial ketohexokinase-dependent fructose metabolism in db/db mice improved mitochondrial homeostasis, synaptic structure, CA1 physiology and cognition (183). Diabetes-associated microglial lactate accumulation has also been linked to H4K12 lactylation and FOXO1-PGC-1α-dependent oxidative stress, implicating lactate-linked signalling in an immunometabolic and epigenetic programme (198). In non-diabetic neuroinflammation, microglial reprogramming altered the local lactate-to-glucose environment while oxidative compensation maintained neuronal gamma activity (165). Conversely, physiological microglial signalling can promote vascular GLUT1 expression and neuronal energy support during activity (199). Microglia are therefore neither constitutive competitors nor passive bystanders. Direct evidence that diabetic microglia capture substrate at the expense of neuronal carbon entry remains absent.

Lactate further shows why abundance cannot identify destination. Chronic streptozotocin diabetes produces progressive hippocampal lactate accumulation and partial behavioural improvement after LDH-sensitive intervention (200), whereas acute exogenous lactate can improve recognition memory and sharp-wave-ripple amplitude in diabetic mice (172). During hypoglycaemia in type 1 diabetes, a much larger brain lactate pool was not accompanied by greater lactate-derived glutamate or glutamine labelling (9). These findings are compatible with chronic accumulation and acute network rescue coexisting. They do not identify the endogenous source, recipient cell, oxidative fate or ATP contribution of lactate.

Compensation and subcellular organisation further weaken inference from transporter abundance or regional averages. Partial astrocytic GLUT1 reduction can induce reprogramming that preserves energetics and cognition (201). Local ATP synthesis sustains active synapses (11), while stable dendritic mitochondrial compartments support nearby translation during plasticity (46). Neuronal GLUT3 deletion can reduce terminal glucose and ATP sensor signals despite comparatively preserved somatic measures and bulk FDG uptake (48). Outside diabetes, inflammatory-cell glucose consumption can also mask parenchymal deficits in regional FDG signals (202). Allocation must therefore be resolved at the relevant cellular and subcellular scale.

Current evidence supports substrate-, region- and cell-programme-dependent reorganisation of cerebral carbon handling in diabetes. Tracer models provide the strongest evidence for reciprocal neuronal and astrocytic remodelling, while endothelial and microglial interventions provide cell-associated causal bridges. However, no study aligns cognitive demand, cell-specific carbon flow, energetic state, neural computation and behaviour. Misallocation therefore remains a testable mechanism rather than an established universal or zero-sum phenotype.

### 6.4 Malutilization: substrate-oxidation-function uncoupling

Malutilization begins where delivery ends. It denotes failure to convert an available substrate into useful oxidative and functional energy support, rather than reduced abundance or uptake alone. A molecule may enter brain tissue or exchange rapidly without reaching the mitochondrial matrix, engaging pyruvate dehydrogenase and the tricarboxylic acid cycle, driving coupled phosphorylation, or supplying ATP locally. The phenotype therefore differs from blunted recruitment, mistiming and misallocation, although all four may coexist. A defensible claim must identify the impaired conversion step and connect it to an energetic or functional consequence.

Normal activity recruits glycolysis, oxidative phosphorylation, local ATP synthesis and mitochondrial calcium-dependent regulation across workloads and compartments (11, 15, 72, 85, 86, 94). Static ATP or PCr cannot establish preserved turnover or delivery because high-energy-phosphate pools may remain stable while ATP-related flux changes (16).

Human studies reveal several dissociations, although none closes the pathway in clinically characterised DCI. Acute lipid-induced insulin resistance was associated with a lower hippocampal PCr/ATP ratio during sustained cognitive activity without measuring glucose oxidation or ATP synthesis (17). In type 1 diabetes, transient insulin deprivation creates systemic hyperglycaemia and ketosis while cerebral high-energy-phosphate measures, connectivity and selected cognitive functions deteriorate (203). Conversely, during hypoglycaemia with [3-^13^C]lactate infusion, a markedly larger calculated brain lactate pool was not accompanied by greater lactate-derived glutamate or glutamine enrichment (9). This supports a pool-use dissociation, not reduced absolute lactate oxidation. Other 13C studies found increased acetate transport and oxidation or preserved model-derived tricarboxylic-acid-cycle activity during hypoglycaemia (155, 156). Beta-hydroxybutyrate also contributed more directly to brain metabolism in type 1 diabetes, while medium-chain triglycerides supported working memory during clamped hypoglycaemia (161). Cerebral fuel use is therefore substrate and challenge specific.

Diabetic animal models provide stronger evidence for downstream energetic constraints. In chronically diabetic rats, hippocampal glucose accumulation coexisted with lower NAD+ availability, impaired mitochondrial respiration and reduced respiratory reserve. Nicotinamide mononucleotide improved respiration, neuronal survival and memory without substantially correcting systemic metabolic abnormalities (184). In streptozotocin-diabetic mice, cyclophilin D deletion protected mitochondrial respiration, ATP synthesis, long-term potentiation and memory (176). Manipulating GSK3β–Drp1 signalling likewise ameliorated complex-I, ATP and synaptic defects (175). High-fat-diet-induced insulin resistance reduced mitochondrial ATP-production capacity and oxidative-enzyme activity, which improved with exercise or metformin (185). Insulin withdrawal also reduced cerebral mitochondrial ATP production despite increased circulating monocarboxylates and MCT transcripts, with partial reversal after intranasal insulin (186). These findings support mitochondrial constraints downstream of substrate availability, but isolated-organelle capacity and behavioural rescue do not reconstruct an unmeasured task-evoked flux pathway.

Tracer studies indicate regional and cell-specific reconfiguration rather than uniform loss of use. Goto-Kakizaki rats show lower modelled neuronal tricarboxylic-acid-cycle activity with greater astrocytic activity (180), whereas db/db mice show substrate-specific labelling patterns (181). Hyperpolarised pyruvate signals vary by stage and region (194, 195), and experiments targeting pyruvate entry show that carbon reaching pyruvate or lactate need not enter mitochondrial oxidation or sustain presynaptic function (44, 204).

Lactate highlights the same inferential problem. Chronic streptozotocin diabetes enlarges hippocampal lactate pools and partially responds to LDH-sensitive intervention (200). Fibroblast growth factor 21 reduces lactate accumulation while increasing MCT2, LDHB activity, NAD+, ATP and memory, but did not directly measure lactate uptake or oxidation flux (187). Exogenous lactate can improve sharp-wave-ripple amplitude and recognition memory in diabetic mice (172). Recurrent-hypoglycaemia studies further show that increased lactate transport contributes little additional oxidation even when lactate preserves glucose metabolism and neuronal function (159). Lactate also modulates NMDA signalling and plasticity-gene expression through redox-dependent mechanisms (79). Rescue under these conditions does not identify an endogenous substrate deficit or prove ATP-mediated rescue.

Countervailing findings preclude a unitary failure model. Despite reduced glucose-derived labelling, db/db brain slices increase acetate and beta-hydroxybutyrate use, while isolated mitochondria show greater oxygen consumption and ATP-synthesis capacity (162). These adaptations may preserve energetics, but they do not establish adequate ATP delivery during cognition. Lactate can increase oxygen consumption while high-demand gamma activity and synaptic transmission deteriorate (164). During moderate neuroinflammation, altered local substrate conditions were instead compensated by greater oxidative metabolism that maintained gamma oscillations (165). Oxidation may therefore be insufficient, compensatory or effective according to location, timing, coupling efficiency and workload.

The evidence supports several possible breaks between substrate availability and function, but not a uniform failure of cerebral fuel use. Malutilization remains a substrate-, compartment-, stage- and demand-dependent phenotype until task-locked carbon fate, energetic state and function are linked within one design (**Table 2**).

### 6.5 Disease stage, metabolic history, and reserve exhaustion

The stress-test framework is intended as a common experimental scaffold rather than a prediction that diabetes types, cognitive phenotypes or disease stages share the same challenge-response pattern. In type 1 diabetes, the available human evidence is weighted towards insulin treatment, recurrent hypoglycaemia, impaired awareness and alternative-substrate adaptation, whereas type 2 diabetes evidence more often reflects insulin resistance, obesity, vascular dysfunction and altered task- or insulin-evoked recruitment. These contexts may shift the operating point, the substrate available for recruitment and the node at which failure becomes detectable. Cognitive phenotype is likewise relevant because memory, executive, attentional and processing-speed deficits engage partially different circuits and may therefore expose different regional or temporal vulnerabilities. Disease stage may further alter the direction of the observed response: preserved or increased recruitment can coexist with vulnerability at one stage, whereas blunted, mistimed or inefficient responses may emerge under greater demand or accumulated metabolic burden. These possibilities should not be interpreted as fixed sequential stages or diabetes-type-specific signatures; the framework instead requires challenge-response patterns to be defined within the relevant diabetes type, cognitive domain, metabolic history and disease context.

Neurometabolic phenotypes in diabetes are contingent rather than fixed. They reflect disease duration, cumulative glycaemic exposure, treatment, antecedent hypoglycaemia, vascular burden, substrate availability and imposed demand. Metabolic reserve is operationally the additional delivery, pathway flux and energetic output recruitable above baseline within a physiologically relevant window, together with post-challenge recovery. Reserve exhaustion would require progressive contraction of this increment that predicts neural or cognitive failure. No study has demonstrated this trajectory end to end in clinically characterised DCI. The concept remains an interpretive model, not a synonym for low resting metabolism or an established natural history.

Early diabetes does not shift cerebral metabolism uniformly. During euglycaemic hyperinsulinaemia, lower peripheral insulin sensitivity was associated with greater brain FDG uptake (27), whereas adults with prediabetes or early type 2 diabetes showed lower resting metabolism in Alzheimer-vulnerable regions, diffuse encoding responses and poorer delayed recall (123). These distinct operating conditions preclude a simple hypermetabolism-versus-hypometabolism contrast. After shorter high-fat-diet exposure, increased hyperpolarised pyruvate-to-lactate exchange preceded differences in the selected water-maze outcomes; with longer exposure, it coexisted with reduced 13C delivery, regional pyruvate-dehydrogenase suppression and memory impairment (194). Streptozotocin diabetes also produced stage-dependent glucose- and acetate-labelling patterns (205), whereas an intermediate stage showed enlarged high-energy-phosphate pools with largely preserved cognition (206). Each pattern is compatible with reconfiguration, but none quantifies spare capacity.

Longitudinal evidence supports asynchronous, partly reversible change rather than a single decline. Serial MRS studies show stage- and region-specific metabolite trajectories in streptozotocin and db/db models (207, 208), including progressive hippocampal lactate accumulation (200, 209). Other staged observations include loss of insulin-mediated microvascular recruitment after prolonged high-fat feeding (138) and an association between two-year perfusion decline and functional deterioration (210). These studies establish time dependence, but most did not repeat the same demand-evoked flux across time.

Late-stage findings nevertheless identify plausible reserve limits. Diet-induced diabetes reduced hippocampal glycogen and abolished its net response to local insulin, although one endpoint cannot resolve synthesis, breakdown or task-related mobilisation (19). High-fat-diet-induced insulin resistance lowered mitochondrial ATP-production capacity and oxidative-enzyme activity in insulin-responsive regions (185), whereas chronic experimental diabetes can reduce NAD+ availability, respiratory reserve and memory (184). Modelled ^13^C data in Goto–Kakizaki rats also indicated lower neuronal tricarboxylic-acid-cycle activity and glutamate–glutamine cycling (180). Counterevidence includes db/db brain slices that increased acetate and ketone use while isolated mitochondria showed greater oxygen consumption and ATP-generating capacity (162). Brain glucose responses to matched hyperglycaemia improved with better glycaemic control (98), and Akita mice retained, and sometimes prolonged, cortical neurovascular responses during progression (169). Reserve exhaustion is therefore neither universal nor necessarily irreversible; it depends on substrate, compartment, challenge and stage.

Current glycaemia cannot fully represent disease state. Over 32 years in DCCT/EDIC, cumulative glycaemic exposure, severe hypoglycaemia and vascular risk independently contributed to later cognitive decline in type 1 diabetes (211). Longer type 2 diabetes duration and poorer control predicted faster decline in Whitehall II (212), whereas midlife diabetes was associated with greater decline over two decades in ARIC (3). Decade-long time-weighted HbA1c was associated with later executive decline and brain-volume loss (213), and visit-to-visit glucose and HbA1c variability predicted Alzheimer disease risk beyond mean exposure (214). These associations do not establish a persistent neuronal metabolic programme and may reflect vascular injury, comorbidity, treatment or reverse causation. A four-year cohort found moderate but not accelerated decline (215), while intensive glucose lowering in ACCORD MIND altered brain-volume loss without improving its primary cognitive outcome (216). Glycaemic history shapes risk and response but is not a deterministic measure of cerebral reserve.

Antecedent hypoglycaemia provides strong evidence that metabolic history can reset subsequent responses. Recent exposure attenuates autonomic, symptomatic and glucose-defence responses, while sustained avoidance can partly restore awareness (217–219). Cerebral glucose responses vary with awareness and prior control, although repeated clamps do not consistently alter modelled transport kinetics (146, 147). Across rodent and human studies, recurrent hypoglycaemia alters task-associated glucose dynamics and context-dependent use of acetate, lactate and glycogen (9, 142, 155, 156, 159, 220–222). These adaptations demonstrate history-dependent resetting, not longitudinal exhaustion of cognitive neurometabolic reserve.

Adaptive recruitment may preserve function at one workload while narrowing the margin at another. The most defensible trajectory is therefore non-monotonic: diabetes may shift the operating point, redistribute activity or recruit alternative substrates before overt failure, followed by stabilisation, further contraction or partial recovery (149, 161, 223). Only a progressive loss of challenge-evoked energetic increment that precedes and predicts functional failure should be termed reserve exhaustion.

## 7 Testing activity-dependent neurometabolic failure in DCI: a falsifiable research agenda

Testing activity-dependent neurometabolic failure requires a staged strategy that moves from phenotype definition to temporally aligned measurement, source–recipient–fate resolution, causal perturbation and longitudinal validation (**Figure 5**). Mechanistic support requires verified engagement of the predicted metabolic node within the relevant challenge window and evidence that downstream function depends on that node, whereas reserve exhaustion can be established only through repeated within-person assessment of dynamic response trajectories.

**Figure 5:**
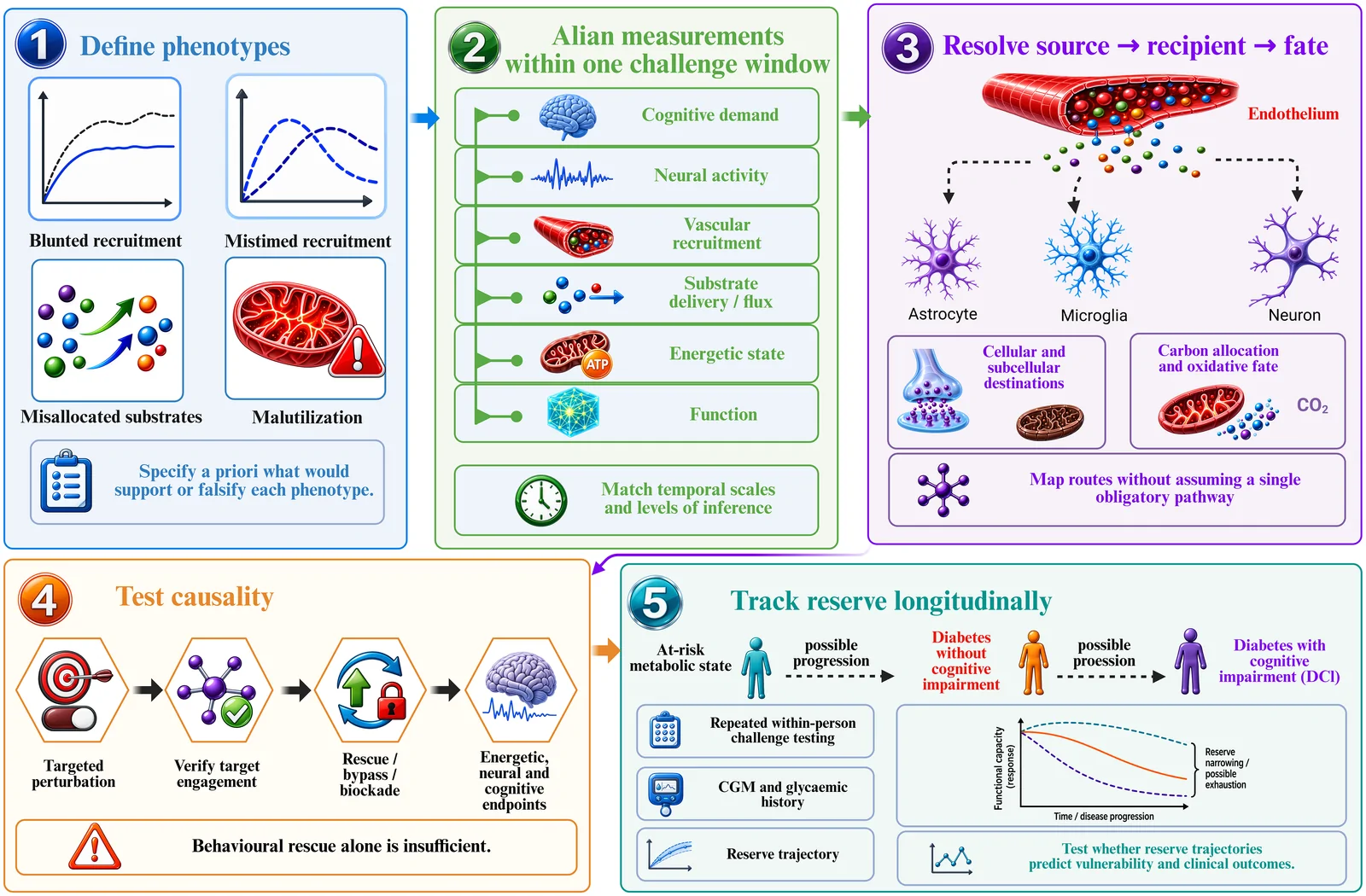
A falsifiable research agenda for testing activity-dependent neurometabolic failure in diabetes-associated cognitive impairment.

### 7.1 Operationalizing the four phenotypes as discriminating predictions

A useful mechanistic phenotype must distinguish the proposed abnormality from plausible alternatives. The labels are assigned to the response dimension and support node directly measured; they are not mutually exclusive causal diagnoses. A challenge may support more than one phenotype at different nodes, and a downstream defect may secondarily alter an upstream response. Causal ordering therefore requires temporally aligned upstream and downstream measurements or targeted perturbation. Each challenge should prespecify demand, neural engagement, baseline state, the relevant compartment and the response dimension being tested. Because transporter abundance, metabolite concentration, pathway rate, high-energy-phosphate turnover and neural function occupy distinct inferential levels (80, 95, 96, 107), every phenotype requires a defined node, a positive criterion and an observation that would refute it.

Blunted recruitment denotes reduced response gain at a specified node after demand has been matched. Cognitive demand produces graded hippocampal glucose responses, while neural activity recruits local ATP synthesis and alters cerebral ATP metabolic rate (10, 11, 16). A low resting value cannot establish blunting. Preserved challenge-evoked amplitude and input-output slope, after accounting for neural engagement, systemic substrate supply and baseline floor or ceiling effects, argue against blunting at the measured node, although failure may remain downstream.

Mistimed recruitment is a kinetic misalignment. Delayed onset, a displaced peak, premature termination or incomplete recovery may preserve peak amplitude or the integrated response yet miss the neural event requiring support. Neurovascular signals evolve across vascular compartments and have intrinsic temporal transfer characteristics (50, 63, 69), while rigid haemodynamic models can convert latency differences into apparent amplitude effects (23). Testing therefore requires a common event clock, adequate sampling and independent neural timing. Equivalent onset, peak latency, duration, recovery and phase relation argue against mistiming.

Candidate substrate misallocation requires three observations: adequate aggregate substrate input or availability, reduced allocation to the task-relevant cell, subcellular compartment or biochemical fate, and compromised energetic or functional support at that site. Cell-resolved uptake and carbon-fate measurements can distinguish parallel routes (15, 48, 71, 77), but current DCI studies do not satisfy all three conditions. Evidence of a reciprocal destination for displaced carbon would strengthen the inference. Redistribution without functional compromise should not be classified as misallocation. Preserved input-corrected allocation, or an apparent shift explained by precursor dilution, pool size, cell composition or regional averaging, argues against misallocation.

Malutilization occurs after substrate delivery: entry is relatively preserved, but oxidation, ATP support or neural output is disproportionately impaired. Lactate accumulation without greater oxidation illustrates why availability cannot be equated with productive use (9), whereas perturbing mitochondrial pyruvate transport links a downstream bottleneck to oxidative ATP failure and impaired presynaptic function (44). Testing must pair entry with oxidation or energetic state and an independent neural endpoint. A deficit explained by insufficient upstream supply should not be assigned to malutilization. These four phenotypes may coexist at identified nodes; reserve exhaustion instead denotes longitudinal contraction of recruitable capacity. **Table 3** links these discriminating criteria to current direct observations, indirect support, major competing explanations and the minimum measurements needed to distinguish each candidate phenotype.

**Table 3:** Discriminating criteria linking candidate neurometabolic failure phenotypes to current evidence and competing explanations.

Candidate phenotype	Direct observations	Indirect support and limiting evidence	Major competing explanations and overlap	Minimum measurements needed to discriminate
Blunted recruitment	Reduced challenge-evoked response gain at a specified support node relative to effective neural demand. Smaller task-evoked cerebrovascular responses and smaller hyperglycaemic brain-glucose pool responses have been observed at selected human nodes (97, 98, 100).	Demand-associated energetic and regional insulin-response abnormalities provide indirect support (17–19, 137, 138). Preserved peak flow, increased insulin-stimulated FDG uptake and greater task activation argue against a universal blunted phenotype (20, 24–30, 130, 169).	Reduced neural engagement; altered systemic substrate or physiology; baseline floor or ceiling effects; vascular limitation; mistiming; or downstream malutilization that secondarily reduces recruitment.	Graded challenge; achieved performance; independent neural-engagement measure; systemic substrate and physiology; baseline-referenced, time-resolved response at the claimed node; downstream energetic or functional readout when causal sufficiency is claimed.
Mistimed recruitment	Altered onset, peak latency, duration or recovery relative to the neural event requiring support. Human and animal studies show delayed, abbreviated or prolonged haemodynamic responses, sometimes with preserved peak or integrated magnitude (20–22, 125, 169, 179).	Phase-specific neural and network abnormalities are compatible with temporal vulnerability but do not establish metabolic mistiming (132, 171, 189).	Altered neural timing; modality-specific delay; vascular transit or reactivity; rigid response modelling; anaesthesia, stimulus or systemic physiological differences.	Shared event clock; independent neural timing; time-resolved vascular or metabolic readout; flexible response modelling; modality-specific delay correction; onset, peak, duration and recovery sampling.
Candidate substrate misallocation	No study currently demonstrates task-locked carbon diversion that both preserves aggregate availability and compromises support of the active compartment in clinically characterised DCI.	Tracer modelling, ex vivo substrate studies and cell-associated perturbations support compartmental metabolic reorganisation (162, 180–183, 194, 195).	Altered delivery; precursor enrichment; dilution; pool-size differences; cell composition; regional averaging; or adaptive redistribution without functional cost. Redistribution alone is not misallocation.	Controlled precursor input; cell- and compartment-resolved carbon fate; task-relevant neural, energetic or functional readout; preferably a reciprocal destination or carbon-balance-consistent account of displaced carbon.
Malutilization	Relatively preserved substrate entry or availability with impaired downstream oxidation, energetic state or neural function during the same challenge. A larger lactate pool without greater lactate-derived labelling is a direct pool–use dissociation, but not a complete cognitive-challenge phenotype (9).	Mitochondrial and functional perturbation studies support downstream constraints (44, 175, 176, 184–187), whereas preserved or increased alternative-fuel use and mitochondrial capacity limit universal claims (156, 162).	Upstream delivery failure; lower effective neural demand; substrate switching; compensatory oxidation; assay-capacity effects; or pleiotropic intervention effects.	Input-corrected entry or uptake; downstream oxidation or ATP/redox dynamics; independent neural or cognitive endpoint during the same challenge; perturbation or rescue when causality is claimed.

### 7.2 Aligning cognitive demand, metabolic flux, energetic state, and neural function

Activity-dependent neurometabolic failure is relational: metabolic support must be judged against neural demand during the same biological episode. Resting values, task responses obtained on another day and terminal assays may reveal coexisting abnormalities, but cannot establish demand-energy uncoupling. The strongest designs align demand and neural engagement, a defined metabolic response, energetic state, and neural or behavioural function within one challenge window (80, 107, 224). Simultaneous acquisition is preferable, although matched within-person sessions remain informative when technical constraints preclude it.

The framework is modular rather than all-or-none. A study need not resolve the complete demand-to-function pathway to make a valid node-specific claim. Technical feasibility may legitimately constrain the number of modalities, provided that inference is restricted to the measured node and that major competing explanations are either measured or explicitly acknowledged. The minimum measurements specified in **Table 3** should therefore be interpreted as claim-specific requirements, not as a requirement that every study acquire the entire multimodal chain.

Imposed task load should be specified, achieved performance quantified, and task-relevant neural engagement measured independently where feasible. Together, these measures characterise effective demand but do not constitute a validated measure of energetic cost. Load-dependent task-evoked responses in diabetes vary across cohorts and glycaemic conditions (129–131), so a shared task label does not establish a shared biological demand. A practical design should retain a prespecified fixed-load series and, where feasible, add individually calibrated conditions. Fixed loads test a common external requirement, whereas calibration can express load relative to estimated capacity or target a prespecified performance range. The manipulated parameter, such as set size or retention interval, and sensory-motor requirements should be specified. Evidence outside diabetes shows that expressing load relative to individual working-memory capacity can materially alter apparent group differences in activation (225). Both the absolute workload and achieved performance should therefore be reported. Accuracy alone is insufficient; reaction time and its variability, errors, omissions, completed trials, strategy and fatigue should be documented across the challenge. Performance-matched or correct-trial analyses should complement, rather than replace, the complete load-performance curve. Behavioural and neural summaries should cover the same acquisition window as block-integrated metabolic measurements. Importantly, trial-level behaviour alone does not confer trial-level metabolic resolution.

Neural engagement should be assessed independently of the metabolic or vascular endpoint whenever feasible. Task-relevant EEG or MEG can be used in humans, whereas electrophysiology can provide aligned neural readouts in experimental models (68, 132). Each measure should target the same circuit, task phase and acquisition window as the metabolic endpoint. Conventional BOLD provides complementary spatial information but should not serve as the sole measure of both neural demand and the vascular response under evaluation (68). Cerebrovascular reactivity should also be characterised, because adjustment can alter task-activation associations in type 2 diabetes (101). Blunting should therefore be evaluated from baseline-referenced response curves and response gain across loads, conditional on systemic input and measured neural engagement. A smaller response is a candidate blunting phenotype only when it is disproportionate to effective demand. Energetic and functional readouts are needed to assess whether altered recruitment is associated with insufficient support at the tested workload; preserved performance alone establishes neither intact reserve nor compensation. Conversely, concurrent reductions in neural and metabolic signals remain directionally ambiguous. Reduced neural engagement can lower metabolic demand, whereas inadequate metabolic support can itself constrain neural recruitment (80, 107, 224). Performance and neural activity should therefore be analysed as both demand indicators and possible downstream outcomes, rather than automatically adjusted away.

The metabolic link must also be specified. CBF indexes perfusion, whereas delivery additionally depends on arterial input and transvascular exchange. FDG-fPET integrates delivery, transport and phosphorylation-dependent trapping; tracer kinetics can estimate defined pathway rates; and CMRO_2_ addresses oxidative metabolism. Simultaneous BOLD-fMRI and FDG-fPET can map haemodynamic and glucose-related responses during a shared task (36, 226), yet BOLD overactivation may occur without greater regional glucose metabolism (227). Glucose utilisation, oxygen consumption and CBF can also change by different fractions, even where CBF and CMRO_2_ are approximately coupled under defined conditions (65, 66). Multimodal discordance therefore localises an unresolved link rather than allowing one readout to represent the whole pathway.

Temporal alignment must accommodate each modality’s response function. Electrical activity changes within milliseconds, haemodynamic signals over seconds, and FDG or many spectroscopic readouts over longer integration periods. Calibrated fMRI can estimate task-evoked CMRO_2_ under explicit physiological and modelling assumptions (109, 228–230), whereas inflexible haemodynamic models may misclassify altered latency as altered amplitude (23). Baseline, onset, sustained-demand and recovery windows should be prespecified, with modality-specific delays modelled and both magnitude and kinetics retained.

A feasible minimum human design can be illustrated for blunted cerebrovascular recruitment, for which challenge-based human evidence is comparatively direct (100). Adults with T2D and objectively characterised DCI could be compared with cognitively unimpaired adults with T2D during a prespecified graded working-memory challenge. Essential measurements would include accuracy and reaction time, task-relevant neural engagement assessed by EEG (68, 132), task-evoked middle-cerebral-artery flow-velocity responses measured by transcranial Doppler (100), and concurrent glucose, blood pressure and end-tidal CO_2_, with cerebrovascular reactivity characterised separately (101, 192, 231). The primary test would be the gain of the vascular response across increasing effective demand. Simultaneous acquisition should be used where feasible; tightly matched within-person sessions remain acceptable when technical constraints preclude it, provided that the loss of temporal alignment is acknowledged.

Blunted recruitment would be supported by a reduced vascular response slope despite comparable or explicitly modelled neural engagement, achieved performance, systemic input and cerebrovascular reactivity. The hypothesis would be weakened if the difference were explained by lower neural engagement, baseline or cerebrovascular-reactivity differences, systemic physiology, or a latency shift rather than reduced response gain. Because transcranial Doppler measures arterial velocity rather than regional tissue perfusion, this design would support only node-specific cerebrovascular blunting. Preserved vascular response gain would argue against blunting at this node but would not exclude downstream substrate-delivery, oxidative or energetic failure. The design would not establish causal mediation, reserve exhaustion or therapeutic benefit.

### 7.3 Resolving cell- and compartment-specific carbon allocation

Regional glucose uptake, lactate concentration or ATP content cannot identify cellular recipients or carbon fate. Allocation therefore requires a source-recipient-fate account. Isotopic enrichment informs flux only when precursor input, pool size, sampling kinetics and a suitable model are incorporated (80, 95, 96, 107). Dynamic ¹³C MRS and isotopologue analysis can trace labelled carbon through glycolysis, pyruvate carboxylation, tricarboxylic-acid-cycle turnover and neurotransmitter exchange, but quantitative neuronal and astroglial rates remain model-dependent (232, 233). Misallocation requires reduced task-relevant allocation plus a reciprocal destination or carbon-balance-consistent loss; reduced enrichment alone remains compatible with altered delivery, dilution, efflux or cell composition.

Normal-brain studies caution against imposing one obligatory route on every activity state. Activation can recruit rapid neuronal glucose uptake (71), while cell-targeted measurements support an astrocyte-neuron lactate gradient whose magnitude depends on calibration and intracellular metabolism (77). Other experiments detect neuronal glycolysis without increased lactate uptake (15), and selective interference with neuronal glucose transport or glycolysis impairs network function *in vivo (*48). A DCI-associated shift must therefore be defined relative to a challenge, circuit and carbon fate; transporter abundance in one cell class cannot show carbon capture at another cell’s expense.

Cell-targeted sensors provide temporal resolution that bulk tracing lacks. Glucose sensors track intra- or extracellular dynamics (234, 235), and lactate sensors report rapid cellular changes (236), and across neuronal and astrocytic pools (77). Their signals depend on affinity, pH, intracellular conversion, calibration and cell volume (237) and report local state rather than carbon provenance or net flux. Combining them with labelled precursors and isotopologue analysis is therefore more informative. Subcellular ATP sensors (238), presynaptic measures of local ATP synthesis (11)and mitochondrial pyruvate transport (44), spatial isotope imaging (239)and single-cell neurometabolomics (240) can further resolve heterogeneity. Their platform-specific limitations, including sensor calibration, sampling delay, cell purity and ex vivo turnover, require appropriate controls and orthogonal validation.

Current diabetes studies resolve only fragments of this chain. Compartmental ¹³C NMR in db/db mice suggests altered neuron-astrocyte cooperation (181), while ex vivo analyses show reconfigured energetics and ketone metabolism (162). In humans, the brain-glucose response to hyperglycaemia is blunted in obesity and type 2 diabetes (97), and lactate can accumulate during hypoglycaemia without greater oxidation of blood-derived lactate (9). These findings support altered substrate handling, not task-specific cellular misallocation. Deuterium metabolic imaging and hyperpolarised ¹³C MRI provide regional substrate-metabolism signals (241, 242). position-specific hyperpolarised tracing adds exchange, redox and downstream labelling information but remains sensitive to delivery and modelling (108). Dynamic FDG-fPET is task-sensitive but cannot resolve later carbon fate or cellular identity (32). Cross-species designs should apply the same challenge, using human imaging for regional timing and animal experiments for cell-targeted fate mapping without borrowing cellular resolution for the human signal.

A minimum test of misallocation therefore requires a quantified precursor input, cellular and relevant subcellular attribution, downstream carbon fate, independent circuit engagement, and a destination or loss route accounting for missing carbon. Preserved input-corrected allocation within adequate sensitivity and model identifiability would refute misallocation despite altered regional pools or transporter expression.

### 7.4 Establishing causality through perturbation, rescue, and mechanistic mediation

A metabolic abnormality accompanying cognitive impairment need not cause it. Diabetes alters vascular reactivity, insulin signalling, inflammation, substrate handling and mitochondrial function in parallel (5). A causal claim requires a directional model linking an exposure or intervention to a prespecified metabolic node, then to energetic or neural function and cognition, while alternative paths remain explicit (243–245). The candidate defect must precede functional failure within the relevant challenge interval, and perturbations should be restricted by cell type, circuit, compartment and time where feasible. Target engagement must be shown at the proposed node, with systemic glucose, insulin, perfusion, arousal and motor performance treated as competing routes (107).

Necessity and sufficiency answer different causal questions. Necessity is supported when selective disruption of a candidate process prevents the normal challenge-evoked energetic response and impairs the corresponding neural function. Activity-resolved ATP imaging has shown that local ATP synthesis is required to sustain presynaptic vesicle cycling (11), and activity-dependent GLUT4 mobilisation supports active synapses (72). Neuronal GLUT3 or pyruvate-kinase deletion tests glucose uptake and glycolysis *in vivo (*48), hippocampal GLUT3 knockdown links glucose uptake to memory acquisition (126), and mitochondrial pyruvate-entry manipulation locates a defect downstream of cytosolic availability (44). These are causal precedents, not evidence for the same lesion in DCI.

Pathway-positioned rescue can order successive steps. Brain glycogen-synthase deletion impairs plasticity and long-term memory (76). Lactate restores memory after astrocytic monocarboxylate-transport disruption but not neuronal MCT2 knockdown (12), while pyruvate or β-hydroxybutyrate can bypass inhibited glycogenolysis or MCT1/4 loss but not deficient neuronal import (47). This epistasis supports downstream energetic sufficiency rather than an irreplaceable role for lactate. Cell-restricted deletion of astrocytic adenosine A2B receptors further illustrates circuit-specific testing of demand sensing and metabolic support (193). Rescue remains ambiguous unless target engagement, energetic recovery and neural output are verified.

Mechanistic mediation cannot be reduced to one statistical indirect effect. Even after treatment randomisation, the mediator is not randomised, and estimates depend on mediator-outcome confounding, exposure-mediator interaction, post-treatment confounders and model specification (243–245). Biological mediation instead requires temporal and experimental convergence: the intervention changes the metabolic node in the relevant window; proximal energetic and neural responses follow; selective blockade attenuates benefit; and a correctly positioned rescue restores it. Perfusion, inflammation and mitochondrial function should remain explicit competing or co-mediating paths.

Diabetes models provide an uneven spectrum of causal resolution. Hippocampal insulin-receptor knockdown impairs plasticity and memory without establishing glucose-flux or ATP mediation (177), whereas myeloid-derived VEGF preserves blood-brain barrier glucose transport and cognition during obesity, consistent with a compensatory response (246). Partial Drp1 normalisation improves mitochondrial and synaptic phenotypes (175), but complete neuronal ablation worsens injury (247), making dose and timing decisive. Cyclophilin-D deletion connects mitochondrial permeability, ATP, plasticity and memory more directly (176). PDK1 augmentation is protective without direct evidence for altered neuronal pyruvate-oxidation flux (248). LDHA inhibition limits hippocampal lactate accumulation and memory loss (200), microglial ketohexokinase deletion restores mitochondrial, synaptic and cognitive phenotypes (183); and brain-endothelial HIF-1α suppression counteracts glycolysis-driven lactate production and DCI (182). These studies move beyond association, but differences in developmental compensation, systemic exposure, challenge timing and direct flux measurement still limit how narrowly the rescued pathway can be assigned.

Broad interventions and human studies offer translational relevance but weaker mediation evidence. Exercise and metformin improve brain mitochondrial function without measuring cognition; insulin manipulation changes ATP-related phenotypes (185). Insulin deficiency and intranasal insulin alter brain mitochondrial ATP-related phenotypes, but lactate and ketone use may represent compensation rather than the primary defect (186), and FGF21 improves lactate-use markers and cognition without testing pathway necessity (187). Metabolic management can normalise brain glucose kinetics without proving cognitive mediation (98), while transient insulin deprivation alters energetics, connectivity and cognition amid systemic changes (203). Intranasal insulin trials report parallel changes in cerebral blood flow and selected functional outcomes (249), while systematic evaluation leaves the perfusion-to-cognition relation unresolved (250). These findings establish manipulability and translational relevance, not a completed metabolic mediation chain.

A minimum falsifiable design combines disease-by-perturbation with rescue or occlusion during the same challenge, verifies the target node, and measures an adjacent energetic response, neural function and behaviour. The model is weakened when energetic correction fails to restore function, benefit survives mediator blockade, or functional recovery precedes metabolic recovery. Accordingly, normalisation of a challenge-response abnormality should not be interpreted as cognitive rescue unless the intervention also improves the relevant cognitive outcome and evidence supports a causal contribution of the targeted node to that benefit (243–245).

### 7.5 Longitudinal stress testing in clinically characterised DCI

The longitudinal approach outlined here is intended to evaluate neurometabolic reserve within this experimental framework; any future diagnostic application would require separate clinical validation. Reserve exhaustion is not a fifth phenotype within one challenge. It denotes longitudinal contraction of the response range recruited above baseline: under matched demand and neural engagement, the same individual shows a smaller response, reaches a ceiling at a lower workload or cannot sustain function. A low response at one visit may instead reflect age, engagement, vascular limitation, treatment, acute metabolic state or measurement error. This distinction is essential in heterogeneous DCI (5) and because resting measurements cannot define activation capacity (80).

Longitudinal epidemiology establishes the importance of stage without demonstrating reserve loss. Midlife diabetes has been associated with greater cognitive decline over two decades (3), and established disease is associated with less favourable trajectories than incident diabetes (251). Dysglycaemia is also linked to cognitive decline, white-matter lesions and brain-volume loss (252, 253), with earlier onset associated with greater dementia risk (254). Metabolic history should therefore include mean exposure, glucose variability (255, 256), recurrent severe hypoglycaemia (257–260), disease duration and treatment. These factors may shift the operating point and adaptive range but do not define one causal route; cognitive impairment can itself increase hypoglycaemia risk (258).

A reserve measure requires a response curve rather than one peak. At each visit, calibrated low- and high-demand conditions should align neural engagement, metabolic recruitment, energetic state and performance. Gain, latency, integrated response, recovery and highest sustainable workload can then be estimated. Challenge-sensitive hippocampal energetic changes after acute insulin resistance establish proof of principle (17), while reduced hippocampal glycogen and insulin-evoked mobilisation in diet-induced diabetes nominate stored substrate as one component of reserve (19), consistent with blunted hippocampal metabolic and cognitive responses to insulin (18). None demonstrates longitudinal contraction in clinically characterised DCI.

Clinical characterisation should include diabetes type, age at onset, duration, treatment, vascular and renal disease, obesity, blood pressure, depression, sleep, relevant medication, domain-level cognition and functional status.Challenge selection and interpretation should therefore be stratified by diabetes type and cognitive phenotype rather than assuming a common reference response across heterogeneous DCI. Memory-dominant, executive-attentional or more global cognitive phenotypes may require different task circuits and readouts, while stage comparisons should distinguish altered operating points from true longitudinal contraction of recruitable capacity. Structural MRI can quantify vascular injury and atrophy, with Alzheimer biomarkers added when mixed pathology is likely. Follow-up task difficulty must be recalibrated to maintain comparable neural demand. Continuous glucose monitoring with ecological cognitive testing already resolves person-specific glucose-performance relationships in type 1 diabetes (261, 262), challenge-locked brain metabolism and neural activity are needed to extend this approach to reserve.

Repeated response-curve testing may help distinguish an inaccessible reserve from a progressively contracting one. Improved glycaemic control was accompanied by restoration of the brain-glucose response in a small within-person type 2 diabetes study (98), yet intensive glucose lowering did not improve cognition in ACCORD MIND (216). Intranasal insulin altered perfusion and selected functional outcomes (249), diet reversal normalised regional metabolites and memory in mice (208), and a DCI-enriched multidomain trial had a neutral primary cognitive result (263). These findings are consistent with plasticity or co-recovery, not expansion of demand-evoked capacity. Failure to recover cannot prove irreversible exhaustion without adequate target engagement, treatment duration and consideration of alternative constraints.

Repeated testing can mimic decline or recovery. Practice effects vary with interval and test form (264–266), and strategy, fatigue or habituation can alter activation independently of capacity. Task-fMRI also has limited individual-level reliability (267). Alternate forms, familiarisation, adaptive difficulty, repeated baselines and reliable-change methods are therefore needed (264, 268), together with standardisation of systemic physiology. Reliability must be established for the actual task and platform: resting ASL flow may be reproducible when task-evoked change is less stable (269), static proton-MRS reproducibility does not guarantee reliable dynamic responses (270), and task-specific FDG quantification requires acquisition and kinetic analysis validated for the intended temporal contrast (33). Reserve exhaustion is supported only when within-person contraction exceeds these errors and persists after matching demand and systemic state.

Three potential uses of the stress-test framework should be distinguished. Mechanistic investigation asks whether a challenge-defined abnormality identifies a causal limiting node. It therefore requires targeted perturbation or rescue with verified target engagement. Prognostic assessment asks whether a baseline response phenotype or its longitudinal trajectory predicts subsequent cognitive decline beyond established clinical risk factors. It therefore requires prospective validation and reproducible measurement. Treatment selection is a predictive-biomarker question and requires a stronger standard. It requires prospective evidence that the baseline stress-test phenotype modifies the effect of an intervention targeting the implicated pathway, ideally through a prespecified treatment-by-phenotype interaction in a randomised trial. None of these clinical applications is currently validated. The present framework should therefore be regarded primarily as a mechanistic and experimental research tool; prognostic and treatment-selection applications remain unvalidated possibilities for future study.

## 8 Conclusion

DCI is commonly interpreted through chronic abnormalities in glucose availability, insulin signalling, vascular function or mitochondrial metabolism. Resting measurements of these processes, however, do not establish whether the brain can coordinate energy supply when neural activity increases. Within this conceptual and experimental framework, cognitive demand is proposed as a neurometabolic stress test to investigate adaptive limitations that may not be apparent from resting perfusion, metabolite concentrations or high-energy-phosphate pools. The relevant question is not simply how much substrate is present, but whether vascular recruitment, substrate delivery, cellular allocation, oxidation, phosphotransfer and neural function are matched in magnitude, timing and location within the same challenge window.

The available evidence supports individual components of this model but does not yet establish an uninterrupted causal chain from cognitive demand to metabolic flux, energetic state, neural computation and cognitive performance in clinically characterised DCI. Current findings are compatible with four non-exclusive response phenotypes: blunted recruitment, mistimed recruitment, substrate misallocation and malutilization. These phenotypes should not be regarded as universal features or discrete disease subtypes. Their expression may vary across brain regions, disease stages and metabolic histories, and several may coexist within the same individual. These within-challenge patterns do not establish reserve exhaustion, which requires repeated within-person evidence of a progressively contracting response range under comparable demand and physiological conditions; one attenuated response is insufficient. Likewise, preserved resting measures cannot exclude reduced neurometabolic reserve, whereas altered metabolite pools cannot, by themselves, identify the underlying transport, turnover or oxidative process.

Resolving these uncertainties will require challenge-locked experimental designs that align cognitive demand with neural activity, vascular recruitment, substrate flux, energetic support and functional output. Cell- and compartment-resolved measurements, combined with targeted perturbation, rescue and longitudinal testing, will be essential for distinguishing causal failure from compensation or downstream consequence. This approach shifts the field from describing static metabolic abnormalities towards testing whether the diabetic brain can mount an appropriately timed and spatially directed energetic response. Activity-dependent neurometabolic failure remains a testable contributor to DCI rather than an established universal mechanism. The proposed neurometabolic stress test provides a conceptual and experimental framework for testing this proposition, not a validated diagnostic, prognostic or treatment-selection tool. Its central question is no longer simply how much fuel is present in the diabetic brain, but whether that fuel can be mobilised, routed and used quickly enough, in the right place, to sustain cognition.
