# Supplementary material for: Cognitive demand as a neurometabolic stress test in diabetes-associated cognitive impairment: from static metabolite pools to dynamic energy responses
Source: Front Endocrinol (Lausanne). 2026 Oct 20;17:1991245. doi: 10.3389/fendo.2026.1991245 (PMC13728930; doi:10.3389/fendo.2026.1991245)
Supplement: Supplementary file 1 [file Table1.docx]

**Supplementary Methods S1**

**Literature search strategies and evidence selection**

# **S1.1 Search approach and retrieval summary**

PubMed was searched iteratively using eight complementary strategies. Medical Subject Headings and title/abstract terms were combined with Boolean operators. Searches Q1–Q7 excluded review articles; Q8 targeted reviews published in 2020–2026 to provide conceptual context. Backward and forward citation tracking supplemented the database searches.

| **Search** | **Focus** | **PubMed hits** | **Records retrieved** |
| --- | --- | --- | --- |
| Q1 | Activity-related neurometabolism in diabetes | 141 | 141 |
| Q2 | Isotope tracing and dynamic metabolism | 73 | 73 |
| Q3 | Network energetics | 431 | 431 |
| Q4 | Human cognitive-task imaging | 52 | 52 |
| Q5 | Substrate transport and reserve capacity | 1,570 | 1,200 |
| Q6 | Neurovascular coupling, blood–brain barrier function and perfusion | 732 | 600 |
| Q7 | Metabolic interventions | 4,349 | 1,000 |
| Q8 | Related reviews and conceptual context | 193 | 193 |

Counts refer to individual searches and may include overlapping records. Retrieval limits were applied to Q5, Q6 and Q7. These figures are search yields, not counts of unique studies, full-text assessments or final inclusions.

# **S1.2 Full PubMed search strategies**

## **Q1. Activity-related neurometabolism in diabetes**

("Diabetes Mellitus"[Mesh] OR diabet*[Title/Abstract] OR "insulin resistance"[Title/Abstract] OR db/db[Title/Abstract] OR streptozotocin[Title/Abstract] OR STZ[Title/Abstract] OR "high-fat diet"[Title/Abstract] OR HFD[Title/Abstract]) AND (cognit*[Title/Abstract] OR memor*[Title/Abstract] OR learning[Title/Abstract] OR hippocamp*[Title/Abstract]) AND ("activity-dependent"[Title/Abstract] OR "activity dependent"[Title/Abstract] OR "cognitive activity"[Title/Abstract] OR "cognitive demand"[Title/Abstract] OR "task-evoked"[Title/Abstract] OR "task induced"[Title/Abstract] OR stimulation[Title/Abstract] OR encoding[Title/Abstract] OR "working memory task"[Title/Abstract]) AND ("energy metabolism"[Title/Abstract] OR neurometabol*[Title/Abstract] OR "metabolic flux"[Title/Abstract] OR glucose[Title/Abstract] OR lactate[Title/Abstract] OR glycogen[Title/Abstract] OR ATP[Title/Abstract] OR "oxygen consumption"[Title/Abstract] OR "cerebral blood flow"[Title/Abstract] OR neurovascular[Title/Abstract] OR perfusion[Title/Abstract]) NOT review[Publication Type]

## **Q2. Isotope tracing and dynamic metabolism**

("Diabetes Mellitus"[Mesh] OR diabet*[Title/Abstract] OR "insulin resistance"[Title/Abstract] OR db/db[Title/Abstract] OR streptozotocin[Title/Abstract] OR STZ[Title/Abstract] OR "high-fat diet"[Title/Abstract] OR HFD[Title/Abstract]) AND (brain[Title/Abstract] OR hippocamp*[Title/Abstract]) AND ("13C"[Title/Abstract] OR "carbon-13"[Title/Abstract] OR hyperpolarized[Title/Abstract] OR "isotope tracing"[Title/Abstract] OR "isotopic tracing"[Title/Abstract] OR "metabolic flux"[Title/Abstract] OR microdialysis[Title/Abstract] OR "31P-MRS"[Title/Abstract] OR "phosphorus magnetic resonance"[Title/Abstract]) NOT review[Publication Type]

**Q3. Network energetics**

("Diabetes Mellitus"[Mesh] OR diabet*[Title/Abstract] OR "insulin resistance"[Title/Abstract] OR db/db[Title/Abstract] OR streptozotocin[Title/Abstract] OR STZ[Title/Abstract] OR "high-fat diet"[Title/Abstract] OR HFD[Title/Abstract]) AND (hippocamp*[Title/Abstract] OR brain[Title/Abstract]) AND (gamma[Title/Abstract] OR "sharp-wave ripple"[Title/Abstract] OR "sharp wave ripple"[Title/Abstract] OR "theta-gamma"[Title/Abstract] OR "long-term potentiation"[Title/Abstract] OR LTP[Title/Abstract]) AND (glucose[Title/Abstract] OR lactate[Title/Abstract] OR ATP[Title/Abstract] OR mitochondri*[Title/Abstract] OR glycogen[Title/Abstract] OR metabol*[Title/Abstract]) NOT review[Publication Type]

## **Q4. Human cognitive-task imaging**

(diabet*[Title/Abstract] OR "insulin resistance"[Title/Abstract]) AND humans[MeSH Terms] AND (cognit*[Title/Abstract] OR memor*[Title/Abstract]) AND (task[Title/Abstract] OR encoding[Title/Abstract] OR "working memory"[Title/Abstract] OR "cognitive activity"[Title/Abstract]) AND (fMRI[Title/Abstract] OR BOLD[Title/Abstract] OR fNIRS[Title/Abstract] OR "arterial spin labeling"[Title/Abstract] OR ASL[Title/Abstract] OR PET[Title/Abstract] OR "31P"[Title/Abstract] OR perfusion[Title/Abstract] OR "cerebral blood flow"[Title/Abstract]) NOT review[Publication Type]

## **Q5. Substrate transport and reserve capacity**

("Diabetes Mellitus"[Mesh] OR diabet*[Title/Abstract] OR "insulin resistance"[Title/Abstract] OR db/db[Title/Abstract] OR streptozotocin[Title/Abstract] OR STZ[Title/Abstract] OR "high-fat diet"[Title/Abstract] OR HFD[Title/Abstract]) AND (cognit*[Title/Abstract] OR memor*[Title/Abstract] OR learning[Title/Abstract] OR hippocamp*[Title/Abstract]) AND (GLUT1[Title/Abstract] OR GLUT3[Title/Abstract] OR GLUT4[Title/Abstract] OR MCT1[Title/Abstract] OR MCT2[Title/Abstract] OR MCT4[Title/Abstract] OR glycogen[Title/Abstract] OR lactate[Title/Abstract] OR mitochondri*[Title/Abstract] OR ATP[Title/Abstract] OR glycolysis[Title/Abstract] OR "glucose uptake"[Title/Abstract]) NOT review[Publication Type]

## **Q6. Neurovascular coupling, blood–brain barrier function and perfusion**

("Diabetes Mellitus"[Mesh] OR diabet*[Title/Abstract] OR "insulin resistance"[Title/Abstract] OR db/db[Title/Abstract] OR streptozotocin[Title/Abstract] OR STZ[Title/Abstract] OR "high-fat diet"[Title/Abstract] OR HFD[Title/Abstract]) AND (cognit*[Title/Abstract] OR memor*[Title/Abstract] OR learning[Title/Abstract] OR hippocamp*[Title/Abstract]) AND ("neurovascular coupling"[Title/Abstract] OR "neurovascular uncoupling"[Title/Abstract] OR "cerebral blood flow"[Title/Abstract] OR perfusion[Title/Abstract] OR "blood-brain barrier"[Title/Abstract] OR BBB[Title/Abstract] OR "functional hyperemia"[Title/Abstract]) NOT review[Publication Type]

## **Q7. Metabolic interventions**

("Diabetes Mellitus"[Mesh] OR diabet*[Title/Abstract] OR "insulin resistance"[Title/Abstract] OR db/db[Title/Abstract] OR streptozotocin[Title/Abstract] OR STZ[Title/Abstract] OR "high-fat diet"[Title/Abstract] OR HFD[Title/Abstract]) AND (cognit*[Title/Abstract] OR memor*[Title/Abstract] OR learning[Title/Abstract] OR hippocamp*[Title/Abstract]) AND (exercise[Title/Abstract] OR insulin[Title/Abstract] OR lactate[Title/Abstract] OR ketone*[Title/Abstract] OR "beta-hydroxybutyrate"[Title/Abstract] OR FGF21[Title/Abstract] OR electroacupuncture[Title/Abstract] OR acupuncture[Title/Abstract]) AND (metabol*[Title/Abstract] OR glucose[Title/Abstract] OR lactate[Title/Abstract] OR glycogen[Title/Abstract] OR ATP[Title/Abstract] OR mitochondri*[Title/Abstract]) NOT review[Publication Type]

## **Q8. Related reviews and conceptual context**

(diabet*[Title/Abstract] OR "insulin resistance"[Title/Abstract]) AND (cognit*[Title/Abstract] OR memor*[Title/Abstract] OR brain[Title/Abstract] OR hippocamp*[Title/Abstract]) AND ("brain energy metabolism"[Title/Abstract] OR neurometabol*[Title/Abstract] OR "energy on demand"[Title/Abstract] OR "metabolic flux"[Title/Abstract] OR lactate[Title/Abstract] OR astrocyte*[Title/Abstract] OR "neurovascular coupling"[Title/Abstract] OR "substrate transport"[Title/Abstract] OR "metabolic reprogramming"[Title/Abstract]) AND review[Publication Type] AND 2020:2026[Date - Publication]

# **S1.3 Evidence-selection principles**

**Primary research.** Studies were prioritised when they addressed human T1D or T2D, clearly described diabetes or insulin-resistance models, or relevant metabolic challenges. Evidence was selected for its contribution to understanding cognitive or neural activity, controlled glucose or substrate exposure, central metabolic or vascular responses, and neural or cognitive outcomes. The population or model, brain region, challenge, measured variable and interpretive limitations had to be identifiable. Particular attention was given to hippocampal and cognition-related networks. Studies were not required to resolve the entire demand-to-function pathway, and selection did not depend on whether findings supported the proposed framework.

**Physiological and methodological context.** Normal physiological and methodological studies provided reference mechanisms and clarified what different measurements could establish. Adjacent-disease and non-diabetic insulin-resistance studies were used as indirect evidence, with their relevance qualified in the narrative. Reviews informed conceptual interpretation but were not counted as original mechanistic findings. Protocols without results informed future research directions rather than conclusions about established mechanisms.

**Use of supporting evidence.** Static metabolite, transporter, perfusion or capacity measurements informed background and candidate mechanisms rather than directly demonstrating task-evoked metabolic failure. Peripheral metabolic changes could not establish cerebral substrate utilisation. Behavioural, network, inflammatory or structural outcomes without central metabolic measurements were not considered sufficient to establish a dynamic metabolic mechanism. Mixed metabolic models were identified explicitly, and unverifiable results or unclear methods were not used to support core mechanistic conclusions. These restrictions concerned the claims supported by a study, not blanket exclusion from all background discussion.

# **S1.4 Search limitations**

This was an iterative, concept-driven search rather than an exhaustive systematic review. Retrieval limits in Q5–Q7 may have restricted coverage. Individual search dates and retrieval-order settings are not reported, and citation-tracking yields are not quantified separately. The counts therefore describe individual database searches rather than a complete screening flow.
